# Discovery of Heparin Mimetic, Potent, and Selective Inhibitors of Human Clotting Factor XIIIa

**DOI:** 10.1021/acsomega.4c04518

**Published:** 2024-07-03

**Authors:** Kayla T. Vu, Srabani Kar, Navneet Goyal, Madhusoodanan Mottamal, Daniel K. Afosah, Rami A. Al-Horani

**Affiliations:** † Division of Basic Pharmaceutical Sciences, College of Pharmacy, Xavier University of Louisiana, New Orleans, Louisiana 70125, United States; ‡ Department of Chemistry, Xavier University of Louisiana, New Orleans, Louisiana 70125, United States; § Department of Medicinal Chemistry, School of Pharmacy, Virginia Commonwealth University, Richmond, Virginia 23219, United States

## Abstract

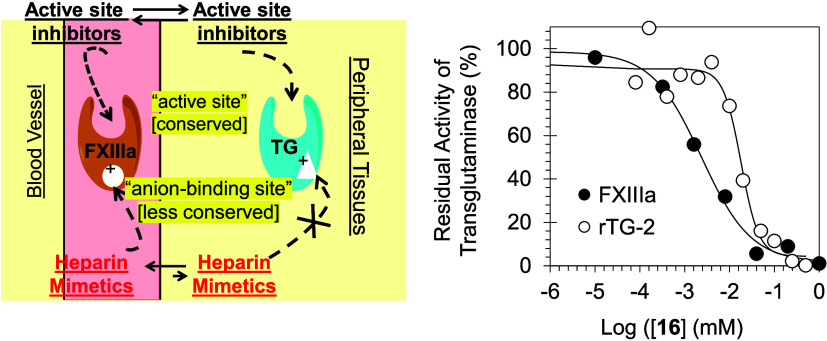

Factor XIIIa (FXIIIa) is a cysteine transglutaminase that catalyzes the last step in the coagulation process. An anion-binding site inhibition of FXIIIa is a paradigm-shifting strategy that may offer key advantages of controlled inhibition. Such an approach is likely to lead to novel FXIIIa inhibitors that do not carry bleeding risks. We previously reported a flavonoid trimer-based allosteric inhibitor of FXIIIa with moderate potency and selectivity. To further advance this approach, we evaluated a series of 27 variably sulfonated heparin mimetics against human FXIIIa. Only 13 molecules exhibited inhibitory activity at the highest concentration tested with IC_50_ values of 2–286 μM. Specifically, inhibitor **16** demonstrated an IC_50_ value of 2.4 ± 0.5 μM in a bisubstrate, fluorescence-based trans-glutamination assay. It also demonstrated a significant selectivity over other clotting factors including thrombin, factor Xa, and factor XIa as well as other cysteine enzymes including papain and tissue transglutaminase 2. Inhibitor **16** did not affect the viability of three human cell lines at a concentration that is 5-fold its FXIIIa-IC_50_. The molecule had a very weak effect on the activated partial thromboplastin time of human plasma at a concentration of >700 μM, further supporting its functional selectivity. Importantly, molecule **16** inhibited FXIIIa-mediated polymerization of fibrin(ogen) in a concentration-dependent manner as shown by the gel electrophoresis experiment. Michaelis–Menten kinetics revealed that the molecule competes with the Gln-donor protein substrate, i.e., dimethylcasein, but not with the Lys-donor small substrate, i.e., dansylcadaverine. Molecular modeling studies revealed that this type of molecule likely binds to an anion-binding site comprising the basic amino acids of Lys54, Lys61, Lys73, Lys156, and Arg244 among others. Overall, our work puts forward a new anion-binding site, selective, nontoxic, sulfonated heparin mimetic FXIIIa inhibitor **16** for further development as an effective and safer anticoagulant.

## Introduction

Venous thromboembolism (VTE) represents a significant public health challenge, ranking as the third most prevalent cardiovascular disorder following coronary heart disease and ischemic stroke. Estimates indicate that VTE contributes to over 100,000 deaths annually in the USA.^1−5^ Despite treatment, a substantial portion of patients, ranging from 30 to 50%, experience recurrent VTE or enduring long-term morbidity.^6−8^ Consequently, the economic burden of VTE is staggering, with annual estimates reaching as high as $69 billion in the USA. Moreover, a bidirectional relationship between VTE and cancer has been substantiated, with cancer patients constituting 15–20% of all VTE diagnoses.^9^ Mechanistically, VTE is initiated by intravascular coagulation activation, leading to the deposition of fibrin, incorporation, and consolidation of red blood cells (RBCs), and the eventual formation of “red thrombi”.^7,10^ Management and prevention of VTE typically involve the administration of anticoagulants, including both indirect anticoagulants (such as unfractionated heparin (UFH), low molecular weight heparins (LMWHs), fondaparinux (FPX), and warfarin) and direct anticoagulants (such as thrombin (FIIa) inhibitors and factor Xa (FXa) inhibitors).^11^

Despite the observed clinical effectiveness of current anticoagulants, they are burdened with significant drawbacks.^12−14^ UFH, for instance, exhibits substantial variability in patient response, necessitating frequent laboratory monitoring. Moreover, heparin-induced thrombocytopenia poses a severe risk associated with heparin therapy. Additional limitations of UFH include the potential development of osteoporosis in patients undergoing prolonged therapy and the heightened risk of contamination with other glycosaminoglycans, which can lead to serious hypersensitivity reactions. The drawbacks of UFH have been mitigated to some extent with the introduction of LMWHs and FPX.^12^ Warfarin, on the other hand, suffers from a narrow therapeutic window and numerous drug–drug and drug-food interactions.^13^ While the safety profiles of newer oral orthosteric inhibitors of thrombin and factor Xa surpass those of heparins and warfarin, challenges persist due to the lack of standardized assays for measuring these drugs in biological fluids, their high cost, and potential contraindications in patients with severe renal dysfunction.^13,14^ Many direct anticoagulants are also substrates of P-glycoprotein, leading to significant drug–drug interaction issues.^15^ Furthermore, nearly all direct anticoagulants undergo hepatic metabolism for elimination, impacting their suitability for patients with hepatic dysfunction.^14^ Importantly, both direct and indirect anticoagulants carry a notable risk of bleeding, particularly intracranial, gastrointestinal, and retroperitoneal bleeding.^16−25^ Thus, the demand for novel anticoagulants remains pressing.

**Figure 1:**
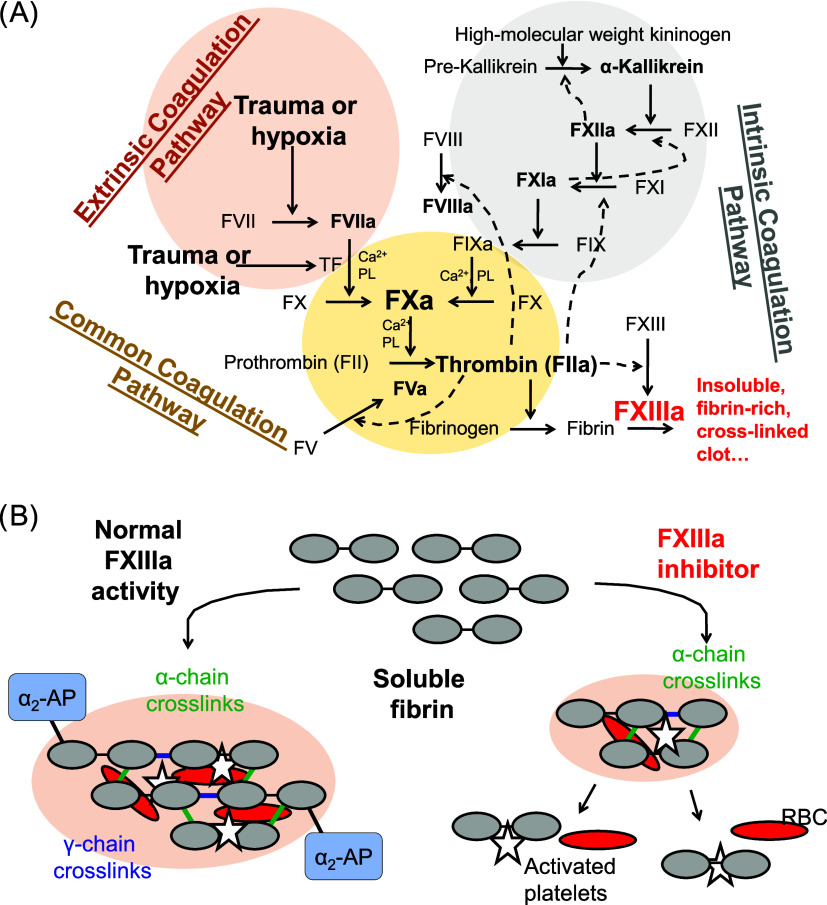
(A) A waterfall representation of the clotting process in which the serine proteases of the intrinsic pathway, the extrinsic pathway, and the common pathway work in a cascade fashion to form a clot that subsequently gets rigidified by FXIIIa-mediated fibrin cross-linking. (B) FXIIIa facilitates the cross-linking of fibrin chains, leading to an increase in the clot size, firmness, and retention of RBCs. Additionally, it plays a role in linking α_2_-antiplasmin to developing thrombi, thereby reducing their vulnerability to breakdown by enzymes like human plasmin. Decreasing or inhibiting FXIIIa activity, notably through inhibitors, can diminish clot size and RBC retention while rendering the clot more prone to degradation by proteolytic enzymes. This has significant implications for the prevention and treatment of venous thrombosis. The white stars represent platelets.

Although exhibiting structural diversity, all existing anticoagulants, whether direct or indirect, primarily target thrombin and/or factor Xa (Figure 1A), both of which are proteases within the coagulation cascade’s common pathway.^11−14^ This mechanism contributes to their effectiveness but also predisposes them to provoke internal bleeding. The objective of this research is to advance the development of novel anticoagulants that are not only efficacious but also safer, offering a reduced risk of bleeding by specifically inhibiting human factor XIIIa (FXIIIa).

With the exception of one, all clotting enzymes are categorized as serine proteases. FXIIIa, in contrast, functions as a transglutaminase and catalyzes the final step in the coagulation cascade, positioning it downstream of thrombin, a pivotal enzyme in both physiological and pathological blood clotting processes (Figure 1A). This distinctive feature has been a subject of scrutiny in the context of venous thromboembolism (VTE). FXIIIa plays a critical role in mechanically stabilizing blood clots by facilitating the cross-linking of fibrin. Additionally, FXIIIa shields the clot from plasmin-mediated fibrinolysis by cross-linking α_2_-antiplasmin to fibrin (Figure 1B).^10,26,27^ Indeed, FXIIIa significantly influences the red blood cell (RBC) content in the clot and clot size. Utilizing a VTE model in the inferior vena cava, thrombi from FXIII-deficient mice exhibited diminished RBC content, reduced weight, and smaller size compared to those from wild-type mice.^28,29^ Intriguingly, mice carrying alanine mutations in fibrinogen residues γ390–396 demonstrated decreased FXIII binding to fibrinogen and delayed cross-linking, mimicking the phenotype of FXIII-deficient mice and producing smaller venous thrombi with reduced RBC content.^28−30^ Moreover, a specific FXIIIa polymorphism was identified to offer substantial protection against both VTE and coronary artery disease.^30^ These findings suggest that the FXIII(a)-fibrinogen axis represents a promising therapeutic target for mitigating VTE. Importantly, neither FXIII-heterozygous nor Fibγ390–396A mice exhibited signs of excessive bleeding.^28−32^

Interestingly, recent FXIIIa synthetic oligopeptide inhibitor delayed clot formation, reduced firmness, and facilitated clot lysis without affecting the clotting times indicating a little impact on hemostasis in whole human blood, determined using rotational thromboelastography.^33,34^ In a rabbit model of VTE, the inhibitor decreased the weight of clots and facilitated flow restoration without prolongation of the bleeding time, which further validated FXIIIa as a novel target for effective and safer anticoagulation.^34^ Overall, an FXIIIa inhibitor will lead to a smaller and “soft” clot that is more susceptible to plasmin hydrolysis, with potentially little impact on hemostasis (Figure 1B). Thus, FXIIIa inhibition is a novel paradigm for developing new effective anticoagulants with a reduced or no risk of bleeding.

**Figure 2:**
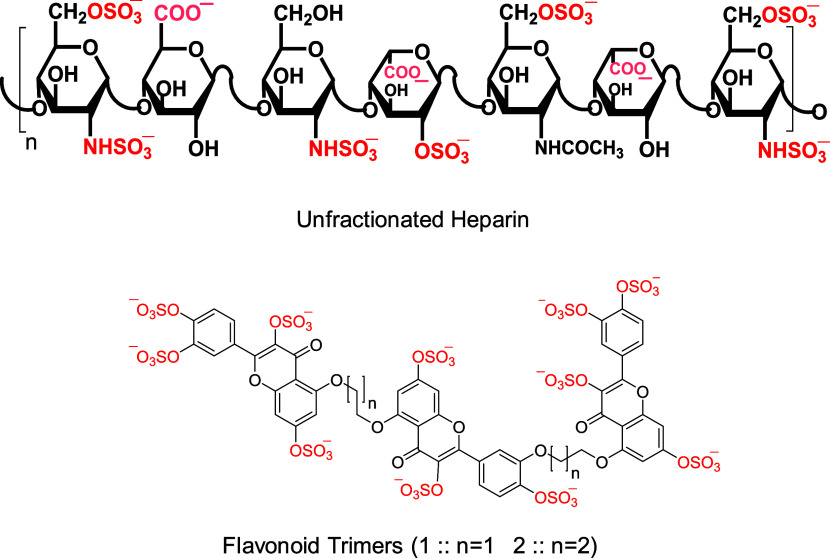
Chemical structures of unfractionated heparin (UFH) as well as previously reported allosteric flavonoid trimer-based FXIIIa inhibitors.

Few FXIIIa inhibitors have been developed thus far.^10^ Nevertheless, those inhibitors bind to the active site of FXIIIa, which is largely conserved among transglutaminases, leading to poor specificity of their function. Interestingly, the crystal structure of FXIII^35^ revealed two potential anion-binding sites that potentially serve as heparin-binding sites. The two sites appear to be less conserved among transglutaminases. Thus, we reasoned that a particular heparin mimetic that electrostatically and hydrophobically complements the putative anion-binding site(s) on FXIIIa may be identified as a selective, allosteric inhibitor. A proof of concept for the allosteric inhibition of FXIIIa by sulfonated flavonoids was previously reported (Figure 2).^36^ Yet, the flavonoid possessed low potency and limited chemical stability.

In this report, we constructed a series of heparin mimetics using synthesized and commercially available molecules to identify more potent and selective nonactive site inhibitors of human FXIIIa. We identified molecule **16** which inhibited FXIIIa with an IC_50_ value of 2.4 ± 0.5 μM. The inhibitor did not affect other clotting factors (thrombin, factor Xa, and factor XIa) at the highest concentration of 150–500 μM. As with other FXIIIa inhibitors, the molecule did not affect the clotting times of human plasma at the highest concentration tested of 705 μM. Michaelis–Menten kinetics revealed that the molecule inhibits FXIIIa by competing with the Gln-donor protein substrate i.e., dimethylcasein, but not with the Lys-donor small substrate i.e., dansylcadaverine, potentially by binding to anion-binding site(s). Inhibitor **16** also inhibited the FXIIIa-induced polymerization of fibrin(ogen) in a concentration-dependent manner. Importantly, inhibitor **16** did not affect the viability of three cell lines indicating its lack of cellular toxicity at a concentration as high as 10 μM. The molecules also exhibited significant selectivity over other cysteine enzymes including papain and tissue transglutaminase-2 (TG-2). The molecule is not expected to distribute to peripheral tissues, and thus, it is expected to demonstrate a higher selectivity over other transglutaminases. Overall, inhibitor **16** is put forward as a potent, selective, nontoxic, and anion-binding site inhibitor of FXIIIa for further development as an effective and relatively safer anticoagulant.

## Results and Discussion

### Rationale for Using Sulfonated Heparin Mimetics to Inhibit Human FXIIIa

Plasma FXIII comprises a heterotetramer consisting of two A and two B subunits. The A subunit houses the catalytic domain, while the B subunit functions as a carrier and regulatory protein. Activation of FXIII occurs through thrombin-mediated hydrolysis of the Arg37-Gly38 bond in the A subunits, followed by the association of Ca^2+^ ions, leading to the dissociation of the A/B subunits. The resultant FXIIIa contains a Cys314 residue within the active site, enabling it to catalyze a transglutaminase reaction. This reaction involves the cross-linking of Lys and Gln residues of fibrin α- and γ-chains, ultimately yielding a three-dimensional, insoluble fibrin network.^26,37^

Interestingly, the A subunit of FXIIIa has a relatively large number of basic residues forming surface-exposed batches of two potential anion-binding sites. A similar site on tissue TG-2 was recently identified as a heparin-binding site and found to be important for enzyme function.^38,39^ Notably, the anion-binding sites of various enzymes exhibit structural diversity, as they vary in the number and distribution of basic residues and hydrophobic subdomains.^38−40^ These structural disparities may contribute to the recognition of specific anionic molecules over others, as different combinations of electrostatic and hydrophobic elements are involved. Consequently, a sulfonated molecule that complements the putative anion-binding site on FXIIIa both electrostatically and hydrophobically may be identified as a selective inhibitor. The feasibility of this approach for inhibiting FXIIIa was previously demonstrated with sulfonated flavonoid trimers (Figure 2B), serving as a proof of concept.^36^

Importantly, these molecules will function mechanistically as anion-binding site inhibitors. Anion-binding site inhibitors provide two key advantages over orthosteric (active site) inhibitors:^41−46^ (i) heightened specificity of function as anion-binding sites on transglutaminases are typically not conserved (Figure S1 in Supporting Information), and (ii) improved regulation of enzymatic efficacy, which may be less than 100% depending on the extent of conformational change in the active site induced by the anion-binding site ligand binding. Consequently, anion-binding site FXIIIa inhibitors potentially outperform orthosteric FXIIIa inhibitors by offering superior selectivity over other transglutaminases, thereby reducing off-target effects. Additionally, FXIIIa inhibition below 100% ensures the preservation of a certain functional level of FXIIIa to maintain hemostasis while preventing bleeding diathesis. This strategy has been previously employed in the development of inhibitors for other clotting factors.^47,48^ Furthermore, the anionic nature of the sulfonated molecules provides several pharmaceutical advantages, including (i) high water solubility, which is anticipated to enhance their utility as anticoagulants; (ii) diminished cellular, fetal, and nervous system toxicity due to their charged nature, which restricts diffusion through physiological membranes/barriers and thus limits distribution to nonvascular tissues (Figure S1); and (iii) enhanced chemical stability compared to other anionic groups (less susceptible to hydrolysis by phosphatases or sulfatases).

### Human FXIIIa Inhibition and Structure–Activity Relationship of Sulfonated Heparin Mimetics

**Figure 3:**
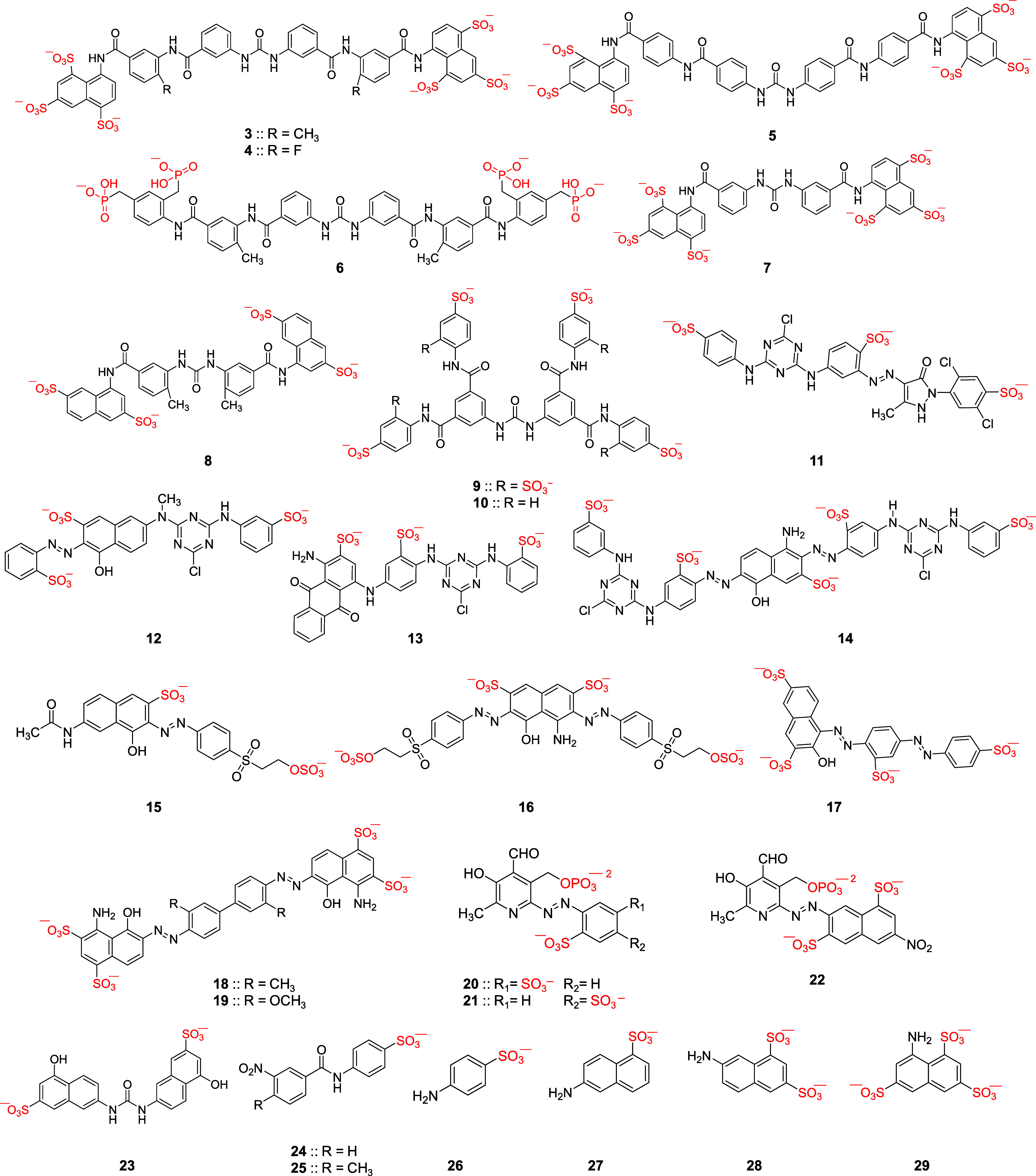
Chemical structures of oligomeric (**3**–**25**) and monomeric (**26**–**29**) sulfonated heparin mimetic molecules investigated in this study as inhibitors of human FXIIIa.

A series of 27 sulfonated molecules (Figure 3) was screened for inhibition of human FXIIIa using a modified bisubstrate, fluorescence-based trans-glutamination assay, as described earlier.^36,49^ Dansylcadaverine and *N*,*N*-dimethylcasein were used as two substrates, which upon FXIIIa-dependence conjugation show a marked increase in fluorescence at 550 nm (λ_EX_ = 360 nm). The sulfonated molecules were structurally diverse given their number of anionic groups (mono- (inhibitors **24**–**27**) to octa- (inhibitor **9**)), number of building blocks (monomeric (molecules **26**–**29**) to heptanoic (inhibitor **14**)), and the nature of the building block of oligomeric derivatives (benzamide derivatives (inhibitors **3**–**10**, **24**, and **25**), triazine derivatives (inhibitors **11**–**14**), and azo-naphthalene derivatives (molecules **15**–**22**), and ureido-naphthalene derivative (**23**)) (Figure 3).

**Figure 4:**
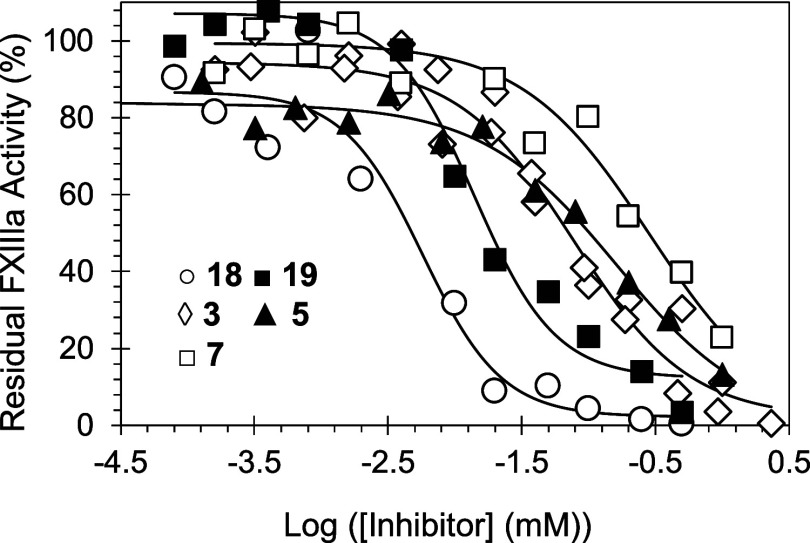
Direct inhibition of FXIIIa by sulfonated molecules (**3**, **5**, **7**, **18**, and **19**). The inhibition potential was investigated using a bisubstrate, fluorescence-based trans-glutamination assay. The solid traces are the sigmoidal data fits using eq 1 to obtain the IC_50_ values, HS, and Δ*Y*%.

To measure the potency and efficacy of these inhibitors, the dose-dependence of FXIIIa inhibition was evaluated using logistic eq 1 (Table 1). The potency of inhibition refers to the IC_50_ (*x*-axis), whereas the efficacy refers to the net change in residual FXIIIa activity (Δ*Y*%) (*y*-axis). Representative inhibition profiles are shown in Figure 4. Iodoacetamide, a nonselective inhibitor of thiol-containing enzymes, was used as a positive control. It inhibited human FXIIIa with an IC_50_ of ∼2.9 μM (Table 1). Results of the trans-glutamination assay indicate that none of the monomeric building blocks (molecules **26**–**29**) inhibited FXIIIa at the highest concentration tested of 200 μM. Likewise, the ureido-naphthalene derivative **23** did not inhibit FXIIIa at the highest concentration tested. Furthermore, oligomeric benzamide derivatives (inhibitors **3**–**10**, **24**, and **25**) exhibited a variable level of inhibition potency with IC_50_ values of 77.2–285.6 μM, and in fact, inhibitors **4**, **8**, and **10** did not inhibit FXIIIa at the highest concentrations tested. The most potent inhibitor in this group is the hexa-sulfonated molecule **3** with an IC_50_ value of 77.2 ± 17.1 μM. Interestingly, this level of potency is similar to the previously reported flavonoid trimers (**1** and **2**). Importantly, the triazine derivatives (inhibitors **11**–**14**) inhibited FXIIIa with better potency as revealed by their IC_50_ values of 3.1–23.6 μM (Table 1). The most potent inhibitor in this group is the trisulfonated inhibitor **13** with an IC_50_ value of 3.1 ± 0.3 μM (Table 1). The azo-naphthalene derivatives (inhibitors **15**–**22**) exhibited considerable inhibition variability. Although molecules **20**–**22** were not active against FXIIIa, other molecules inhibited FXIIIa with IC_50_ values of 2.4–16.6 μM, making them the most potent among all scaffolds evaluated in this report. The most potent inhibitor in this group is the tetra-sulfonated inhibitor **16** with an IC_50_ value of 2.4 ± 0.5 μM (Table 1).

**Table 1:**
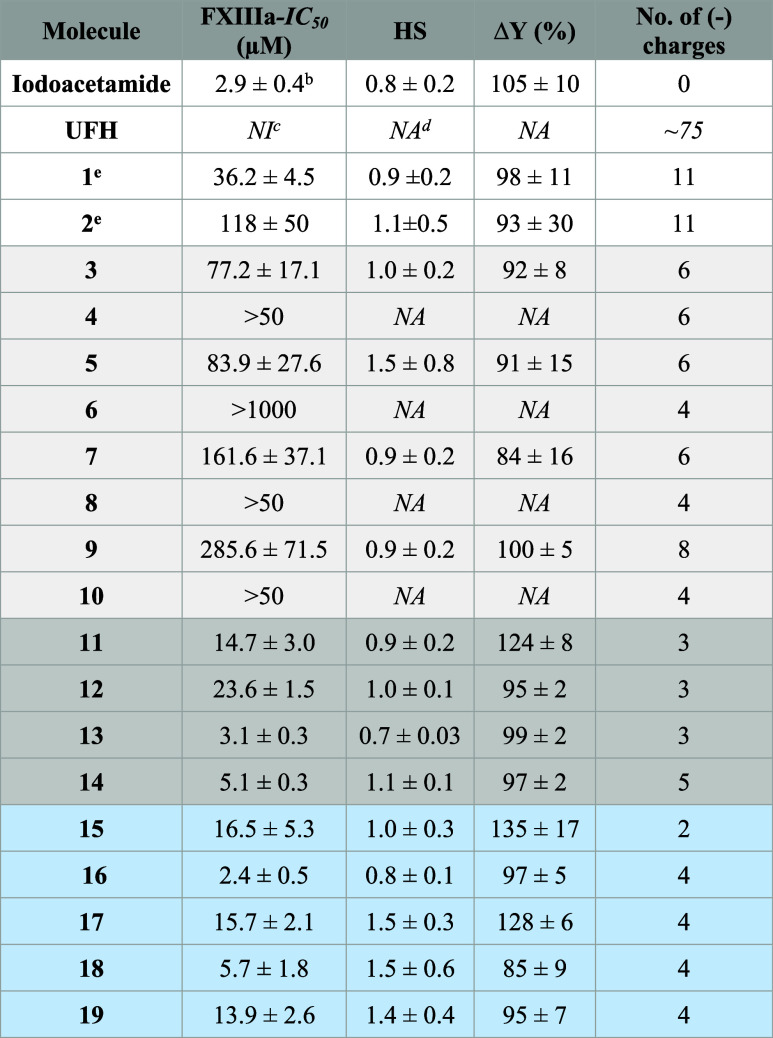
Inhibition of Human FXIIIa by Heparin Mimetics Using Bisubstrate, Fluorescence-Based Trans-glutamination Assay^a^

a The inhibition profiles were obtained following nonlinear regression analysis of direct inhibition of FXIIIa in a suitable buffer.

b Errors represent ±1 SE.

c No inhibition.

d Not available.

e Values reported in *PLoS One* 11(7): e0160189.

Together, comparing the potency of different inhibitors of FXIIIa in Table 1 suggests that inhibiting FXIIIa using sulfonated heparin mimetics can be independent of the number of sulfonate groups and can be optimized by tuning the aromatic core structures. Not only that but also the fact that UFH itself does not inhibit FXIIIa, although it is sulfonated, emphasizes the significance of the aromatic characteristics of potential inhibitors over the alicyclic saccharide backbone. Thus, the most potent heparin mimetic FXIIIa inhibitors are azo-naphthalene derivatives (2.4–16.6 μM), followed by the triazine derivatives (3.1–23.6 μM), followed by the flavonoid trimers (38.2–118 μM), followed by the benzamide derivatives (77.2–285.6 μM), while UFH is not active. Furthermore, it appears that the most optimal number of sulfonate groups on a potential FXIIIa inhibitor can be 3–6 groups. The most potent inhibitor **16** possesses four sulfonate groups whereas the second most potent inhibitor **13** possesses three sulfonate groups. This is consistent with previous studies which indicated that polyanion-binding proteins do not necessarily rely on the high sulfonation level of their ligands but also on the 3-dimensional orientation of the sulfonate groups. For example, factor XIa was found to preferentially recognize globular inhibitors,^50^ while antithrombin interacted better with linear sulfonated molecules.^51^ For FXIIIa, globular structures such as **9** and **10** are either not active or weak inhibitors. It also appears that the optimal number of structural domains is 3–4 blocks. The most potent inhibitor **16** possesses three structural domains, the second most potent inhibitor **13** possesses four structural domains, and all dimeric structures are eventually not active.

Lastly, regardless of the scaffold used, the same level of efficacy (90–100%) has been achieved by all active molecules. It is also worth mentioning that the inhibition studies for the most potent inhibitors (**13** and **16**) were repeated by using different concentrations of FXIIIa, resulting in similar IC_50_ values.

### Selectivity of FXIIIa Inhibition: Inhibition of Related Enzymes, Effect on Clotting Times, and Effect on Cellular Proliferation

One of the motivations for targeting anion-binding sites on human FXIIIa was to achieve selectivity in inhibition over closely related enzymes such as other clotting serine proteases or other transglutaminases and cysteine proteases. To demonstrate this characteristic, the hydrolysis of appropriate small tripeptidic chromogenic substrates of thrombin, factor Xa, and factor XIa, which are three anion-binding proteins with significant roles in coagulation, was assessed at pH 7.4 and 37 °C for five molecules (**13**, **14**, **16**, **18**, and **19**) (Table 2). Evidently, inhibitor **18** was not selective as it inhibited thrombin, factor Xa, and factor XIa at comparable potency with IC_50_ values of 1.2–12.7 μM. Inhibitor **19** demonstrated selectivity indices of at least 7-fold over thrombin and factor Xa, yet it more potently inhibited factor XIa with an IC_50_ value of 6.2 μM. Inhibitor **13** exhibited selectivity indices of 28-fold, 20-fold, and >48-fold over thrombin, factor Xa, and factor XIa, respectively. Inhibitor **14** exhibited selectivity indices of 100-fold, 6-fold, and >30-fold over thrombin, factor Xa, and factor XIa, respectively. Importantly, inhibitor **16** exhibited >208-fold selectivity over thrombin and factor Xa, and >65-fold selectivity over factor XIa.

**Table 2:** Inhibition of Other Human Clotting Factors by Heparin Mimetics Using the Chromogenic Substrate Hydrolysis Assay.^a^

heparin mimetics	FXIIIa	thrombin	FXa	FXIa
**13**	3.1 ± 0.3 μM	86.9 ± 33.6^b^ μM	63.5 ± 13 μM	>150^c^
	0.7 ± 0.03	1.9 ± 1.5	1.5 ± 0.5	
	99 ± 2%	43 ± 11%	87 ± 4%	
**14**	5.1 ± 0.3 μM	>500	30.96 ± 5.70 μM	>150
	1.1 ± 0.1		2.5 ± 0.9	
	97 ± 2%		53 ± 5%	
**16**	2.4 ± 0.5 μM	>500	>500	>150
	0.8 ± 0.1			
	97 ± 5%			
**18**	5.7 ± 1.8 μM	8.2 ± 1.3 μM	12.7 ± 0.7 μM	1.2 ± 0.1 μM
	1.5 ± 0.6	2.6 ± 1.0	1.9 ± 0.2	1.9 ± 0.3
	85 ± 9%	62 ± 5%	80 ± 2%	104 ± 3%
**19**	13.9 ± 2.6 μM	>100 μM	>100 μM	6.2 ± 0.4 μM
	1.4 ± 0.4			1.5 ± 0.1
	95 ± 7%			106 ± 3%

a The inhibition parameters were obtained following nonlinear regression analysis of direct inhibition of human clotting factors in appropriate Tris-HCl buffers of pH 7.4 at 37 °C. Inhibition was monitored by spectrophotometric measurement of residual enzyme activity.

b Errors represent ±1 SE.

c Estimated value was based on the highest concentration of the inhibitor used in the experiment.

**Figure 5:**
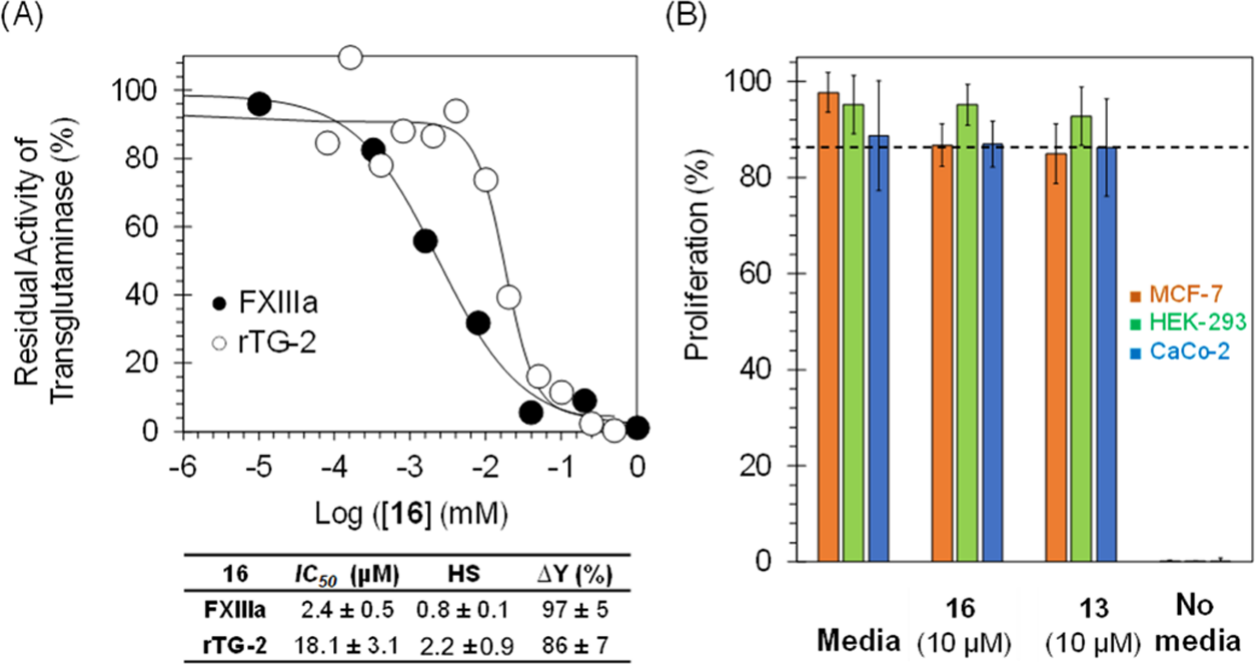
(A) Selectivity of inhibitor **16** toward human FXIIIa and over rTG-2. (B) Effects of inhibitors **13** and **16** at 10 μM (3–5-fold of FXIIIa-IC_50_) on the cell viability of three cell lines: breast (MCF-7), kidney (HEK-293), and intestine (CaCo-2). Similar results were obtained when molecules were tested at concentrations as high as 100 μM.

FXIIIa and TG-2 belong to the same transglutaminase superfamily, and they share similar protein folds with a sequence similarity of 34%. Although we believe that sulfonated heparin mimetics are less likely to distribute to peripheral tissues, we evaluated inhibitor **16**′s potential to inhibit TG-2 (we used recombinant TG-2). As exhibited by Figure 5A, inhibitor **16** demonstrates a moderate selectivity of 9-fold over rTG-2. Likewise, we studied the inhibition of papain, a related cysteine protease, by molecule **16**. Inhibitor **16** did not inhibit papain at the highest concentration tested of 500 μM (>200-fold selectivity).

To further evaluate the selectivity as well as the potential toxicity of inhibitors, we studied their effects on the clotting times of normal human plasma as well as their effects on the proliferation of three cell lines. On the one hand, the inhibitors’ effects on activated partial thromboplastin time (APTT) and prothrombin time (PT) were measured, as described earlier under *in vitro* conditions,^52−55^ using variable concentrations (0–2000 μM) (Table 3). Results indicated that the inhibitors’ concentrations estimated to double APTT and PT are in the high micromolar range. For example, inhibitor **16**′s concentration to double the APTT was 704.6 ± 69 μM (>290-fold of the FXIIIa-IC_50_), suggesting the lack of a significant effect on heparin-binding coagulation proteins such as thrombin, factor Xa, factor IXa, factor XIa, and antithrombin. On the other hand, the antiproliferative properties of inhibitors **13** and **16** were evaluated in three cell lines of breast (MCF-7), intestine (CaCo-2), and kidney (HEK-293) (Figure 5B), as described earlier.^56^ Similar to testing in human plasma, results indicated that 10 μM of the inhibitors (3–5-fold of the FXIIIa-IC_50_) does not significantly affect the proliferation of the above cell lines. Together, inhibitor **16** appears to exhibit significant selectivity indices over other heparin-binding proteins in human plasma and it is not associated with cellular toxicity at the highest concentration tested of 10 μM. Thus, inhibitor **16** displays very good functional selectivity. Overall, inhibitor **16** can be considered for further development as a potent, selective, and nontoxic inhibitor of human FXIIIa.

**Table 3:** Effects on Human Clotting Times^a^

heparin mimetics	APTT_2X_ (μM)	PT_2X_ (μM)
**11**	1102.72 ± 93^b^	>1667
**12**	2067.59 ± 200	ND
**13**	1187.2 ± 175	ND
**14**	583 ± 60	ND
**15**	>1667	ND
**16**	704.6 ± 69	ND
**17**	681.54 ± 65	868.96 ± 91
**18**	224.3 ± 20	497.9 ± 52
**19**	1840.7 ± 88	3073.2 ± 281

a Prolongation of clotting time as a function of the concentration of heparin mimetics in the APTT and PT assays. Presented is the concentration required to double clotting time.

b Error represents ±1 SE.

### Mechanism of FXIIIa Inhibition by Inhibitor **16**

**Figure 6:**
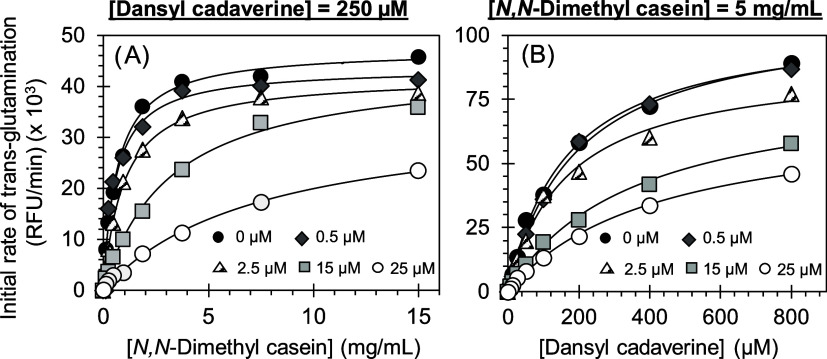
Michaelis–Menten kinetics of dansylcadaverine and *N*,*N*-dimethylcasein conjugation by human FXIIIa in the presence of inhibitor **16**. The initial rate of conjugation at (A) various dimethylcasein concentrations (0–15 mg/mL) and fixed dansylcadaverine concentration (250 μM) or (B) various dansylcadaverine concentrations (0–800 μM) and a fixed dimethylcasein concentration (5 mg/mL) was measured spectrofluorometrically in pH 8.0 buffer at 37 °C. Solid lines represent nonlinear regression fits to the data by the standard Michaelis–Menten eq 2 to yield *K*_M_ and *V*_MAX_. See details in the Materials and Methods.

To elucidate the FXIIIa inhibitory capacity of compound **16**, the kinetics of dansylcadaverine conjugation with dimethylcasein by FXIIIa were investigated with and without the inhibitor using the bisubstrate fluorescence assay.^36,49,55^ At a fixed, high concentration of dansylcadaverine (250 μM), the initial rate of conjugation increased in a hyperbolic manner with increasing concentration of dimethylcasein (0–15 mg/mL) (Figure 6A) in the presence as well as absence of inhibitor **16**. Analysis using the Michaelis equation led to *K*_M_ values for dimethylcasein of 0.65 ± 0.04 mg/mL (∼29.5 μM) and 7.54 ± 0.90 mg/mL (∼342.7 μM) in the absence and presence of **16**, respectively (Table 4). This represents a decrease of ∼11.6-fold in dimethylcasein binding affinity in the presence of **16** at 25 μM. Likewise, *V*_MAX_ also slightly decreased ∼1.4-fold from 47.2 ± 0.8 × 10^3^ RFU/min to 34.9 ± 2.0 × 10^3^ RFU/min in the presence of inhibitor **16** at 25 μM. To derive kinetic constants for dansylcadaverine, we used a fixed, high concentration of dimethylcasein (5 mg/mL) and studied the initial rate of conjugation, as described above. As expected, a hyperbolic profile was observed with increasing concentrations of dansylcadaverine (0–800 μM) (Figure 6B) from which the *K*_M_ and *V*_MAX_ were calculated. The *K*_M_ for dansylcadaverine in the absence of **16** was measured to be 0.20 ± 0.01 mM, which increased in the presence of the inhibitor at 25 μM to 0.42 ± 0.03 mM, representing a ∼2-fold decrease in dansylcadaverine binding affinity (Table 4). Likewise, *V*_MAX_ also decreased ∼1.5-fold from 105.9 ± 3.0 × 10^3^ RFU/min to 69.69 ± 2.9 × 10^3^ RFU/min in the presence of inhibitor **16** at 25 μM. It is important to note that FXIIIa-mediated reaction is a bisubstrate conjugation reaction and its interaction with inhibitor **16** is sensed differently by the two substrates. Whereas the *K*_M_ of the small substrate dansylcadaverine slightly increased (∼2-fold) upon inhibitor **16** binding, it drastically increased (∼11.6-fold) for the larger substrate *N*,*N*-dimethylcasein. Yet, for both substrates, there was a similar slight decrease (∼1.5-fold) in the *V*_MAX_ with the same inhibitor concentration. In these experiments inhibitor **16** was used at varying levels, of which the highest concentration ensured that FXIIIa was essentially fully saturated and inhibited.

**Table 4:** Conjugation of Dansylcadaverine and *N*,*N*-Dimethylcasein by Human FXIIIa in the Presence of Inhibitor **16**^a^

[Dansylcadaverine] = 250 μM		
(16)(μM)	*K*_M_ (mg/mL)	*V*_MAX_ (RFU/min) (×10^3^)
0	0.65 ± 0.04^b^	47.2 ± 0.8
0.5	0.59 ± 0.13	43.5 ± 2.4
2.5	0.97 ± 0.05	42.0 ± 0.6
**15**	3.19 ± 0.30	44.5 ± 1.5
**25**	7.54 ± 0.90	34.9 ± 2.0

[*N*,*N*-Dimethylcasein] = 5 mg/mL		
(16)(μM)	*K*_M_ (mM)	*V*_MAX_ (RFU/min) (×10^3^)
**0**	0.20 ± 0.01	105.9 ± 3.0
**0.5**	0.22 ± 0.02	111.4 ± 4.1
**2.5**	0.20 ± 0.02	89.76 ± 3.8
**15**	0.36 ± 0.04	81.94 ± 4.0
**25**	0.42 ± 0.03	69.69 ± 2.9

a *K*_M_ and *V*_MAX_ values of the two substrates conjugation by human FXIIIa were evaluated using fluorescence spectroscopy (λ_Ex_ = 360 nm and λ_Em_ = 550 nm). RFU indicates relative fluorescence units.

b Error represents ±1 SE.

An alternative way to analyze the data is to evaluate the effect of increasing the concentration of inhibitor **16** on *K*_M_ of the Gln-donor protein substrate (larger substrate: dimethylcasein (DMC)) by plotting *K*_M_ vs [Inhibitor **16**] and on the *V*_MAX_ by plotting 1/*V*_MAX_ vs [Inhibitor **16**] (Figure S2) as well as to study the effect of increasing the concentration of inhibitor 16 on *K*_M_ of the Lys-donor substrate (smaller substrate: dansylcadaverine (DC)) by plotting *K*_M_ vs [Inhibitor **16**] and on the *V*_MAX_ by plotting 1/*V*_MAX_ vs [Inhibitor **16**] (Figure S3). From this analysis, it becomes evident that the most significant effect is that inhibitor **16** decreases the affinity of DMC competitively. Because the binding of large protein substrates (in this experimental condition: dimethylcasein, and in physiological condition: fibrin(ogen)) involves interaction across extended recognition sites, we believe that the interaction of these molecules with FXIIIa is distinct and involves an anion-binding site that does not include the active site Cys.

Overall, the results allude to a unique inhibition mechanism in which, regardless of the substrate binding order, inhibitor **16** appears to better bind to an anion-binding site, and its binding decreases the affinity of the substrates differently, yet it affects the rates of the reaction similarly. In this case, the reduction in the rate of the reaction is likely to be a conformational change in the active site, which most probably carries forward to the subsequent step. This moderate and consistent conformational change is likely to arise from the inhibitor binding to anion-binding site(s). Nevertheless, detailed kinetic evaluation would be necessary to elucidate the parameters of this ping-pong process.

### Effect of Inhibitor **13** and Inhibitor **16** on FXIIIa-Mediated Fibrin Polymerization

**Figure 7:**
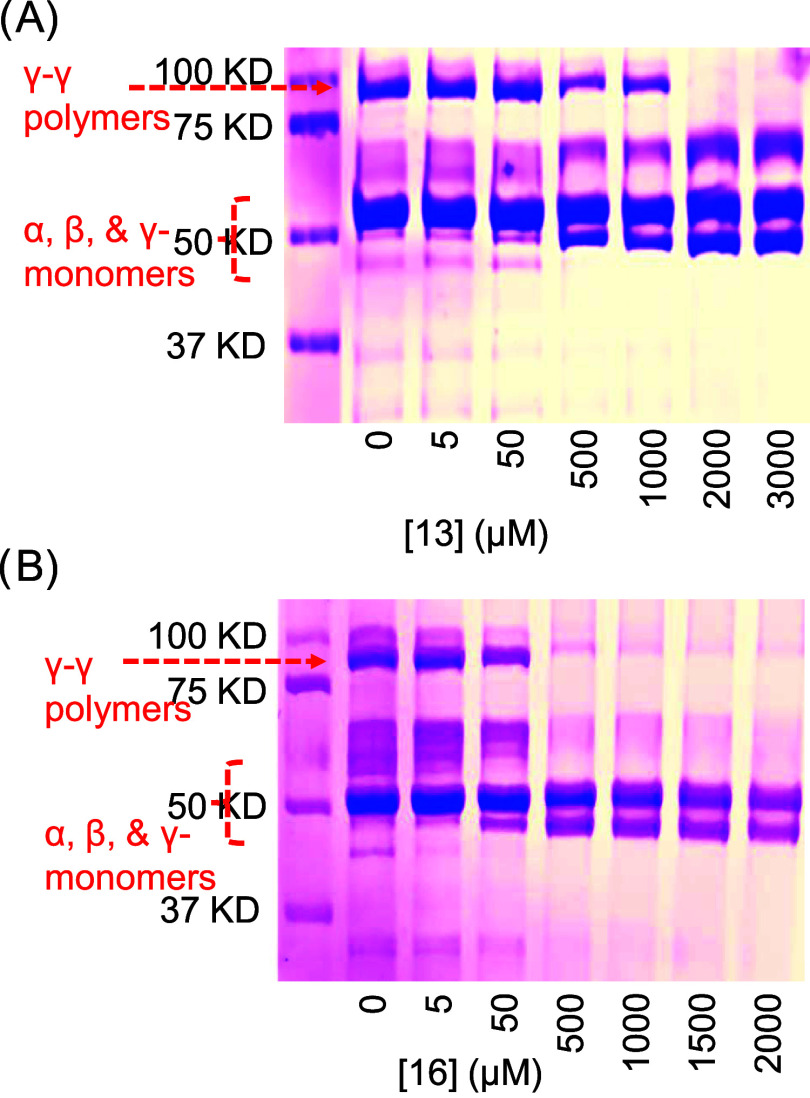
Evaluation of FXIIIa-mediated fibrin polymerization by gel electrophoresis in the presence of (A) inhibitor **13** and (B) inhibitor **16**. The gel electrophoresis experiment shows a dose-dependent effect of inhibitors (5, 50, 500, 1000, 1500, 2000, and 3000 μM) on fibrin cross-linking.

The effect of inhibitors **13** and **16** on FXIIIa-mediated fibrin(ogen) polymerization was investigated by sodium dodecyl sulfate-polyacrylamide gel electrophoresis (SDS-PAGE), as reported earlier.^36,49^ A solution containing 13 mg/mL fibrinogen and 2.0 μg/mL FXIIIa in Tris-HCl buffer of pH 7.4 containing 10 mM CaCl_2_ was either incubated with different concentrations of inhibitors **13** (5, 50, 500, 1000, 2000, and 3000 μM) or **16** (5, 50, 500, 1000, 1500, and 2000 μM) or buffer. The resulting mixture was then clotted in the presence of human α-thrombin (2.5 μg/mL). The clots were incubated for 24 h at room temperature before the addition of denaturing buffer and then incubated overnight at 25 °C. Samples were boiled in a water bath for 10 min before centrifugation at 12 000*g* at 20 °C for 3 min; the supernatants were examined by SDS-PAGE on homogeneous 10% cross-linked gels and stained with Coomassie Brilliant Blue. The first lane contains the protein markers, whereas the second lane contains the cross-linked fibrin(ogen) formed in the presence of human α-thrombin (Figure 7). The lane shows the monomers α-, β-, and γ- bands (∼50–60 kDa) as well as the cross-linked proteins including the lighter γ–γ dimers (∼117 kDa). Figure 7A shows that inhibitor **13** concentration-dependently inhibited the formation of the lighter γ–γ dimers. Likewise, Figure 7B shows that inhibitor **16** concentration-dependently inhibited the FXIIIa-based polymerization of fibrin(ogen) and the formation of the γ–γ dimers, *albeit* at lower concentrations than those of inhibitor **13**, suggesting better potency. In both cases, the inhibition appears to achieve 100% efficacy similar to the inhibition assay results. Overall, the results indicate that the inhibition activity of molecules **13** and **16** toward the catalytic activity of human FXIIIa is physiologically relevant.

### Molecular Modeling Studies

The crystal structure of human FXIIIa with a peptide ligand bound at the active site has been reported.^27^ Mutagenesis and molecular modeling studies have identified the heparin-binding sites on TG-2.^38,39^ FXIIIa and TG-2 belong to the same transglutaminase superfamily, sharing a sequence similarity of 34%. The two heparin-binding sites on TG-2 are mainly comprised of two clusters of basic amino acids. Molecules used in this research contain many negatively charged sulfonate groups and are likely to target clusters of basic amino acids on FXIIIa. By superimposing the crystal structures of TG-2 (1KV3.pdb)^57^ and FXIIIa (4KTY.pdb),^27^ we have identified two clusters of basic amino acids on FXIIIa (labeled here as anion-binding site I and anion-binding site II) that correspond to the two anion-binding sites on TG-2. The basic amino acids that form the anion-binding site I on FXIIIa entail Lys54, Arg56, Lys61, Lys73, Arg244, and Arg303. Likewise, the clusters of basic amino acids that make the anion-binding site II on FXIIIa comprise Arg252, Lys257, Arg260, and Lys269. In addition to these two sites, we included the active site of FXIIIa in our molecular modeling efforts to identify the potential binding site(s) for sulfonated heparin mimetics. The active site was defined by selecting the catalytic triad and the nearby basic amino acids.

**Figure 8:**
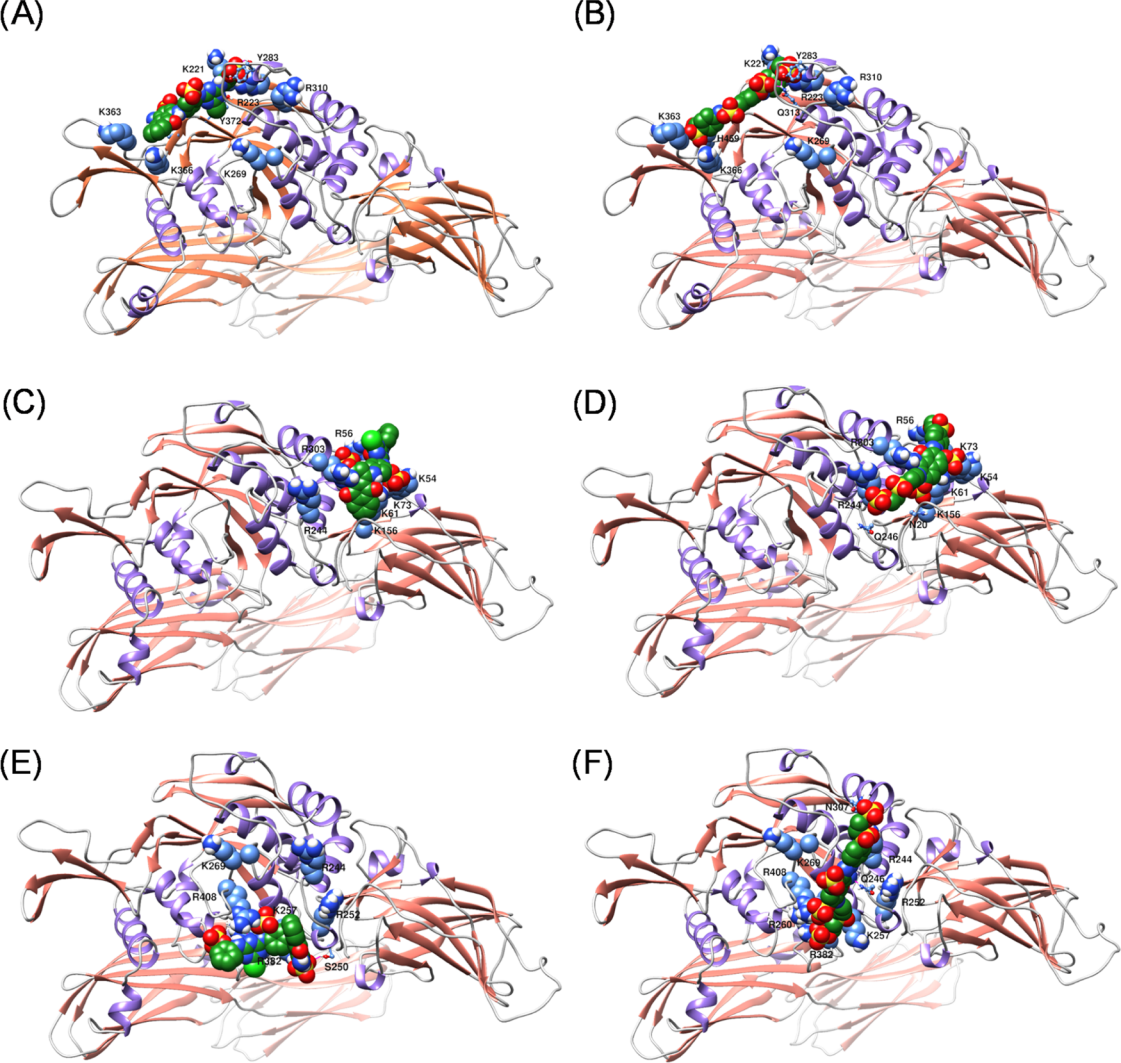
Predicted binding modes of inhibitors **13** and **16** in three different binding sites. Inhibitor **13** is modeled for binding to (A) the Cys-containing active site, (C) anion-binding site I, and (E) anion-binding site II. Inhibitor **16** is modeled for binding to (B) the active site, (D) anion-binding site I, and (F) anion-binding site II. Basic amino acid clusters and nearby important amino acids in each binding site are shown in sphere models.

**Figure 9:**
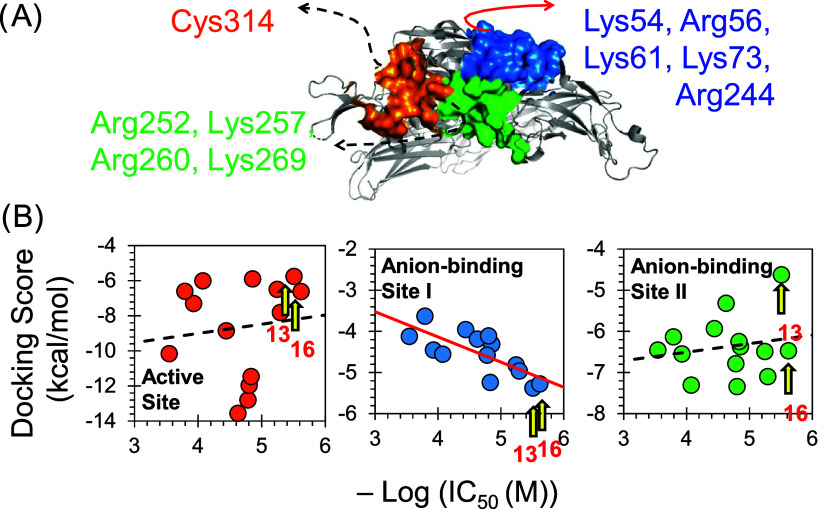
(A) Illustration of the basic amino acids in each of the three potential binding sites on human FXIIIa. (B) Following the docking of the 14 active molecules to the three binding sites and the determination of their FXIIIa inhibition potency, a correlation was established between the docking scores (kcal/mol) and the negative logarithmic value of the corresponding IC_50_ in molar concentration unit.

Figure 8 shows the predicted binding modes of inhibitors **13** and **16** onto three binding sites: Cys-containing active site, anion-binding site I, and anion-binding site II. The binding is primarily driven by interactions (salt bridge and/or hydrogen bonds) afforded by the sulfonate groups to Lys and Arg residues. To elect one of these sites as the most plausible site for binding, we docked the molecules with defined inhibitory activity (with experimentally determined IC_50_) onto the three potential binding sites. Then, we plotted the resulting docking scores presented in kcal/mol (binding energy) against the negative logarithmic value of their molar IC_50_ values (Figure 9). As illustrated, the best correlation is given by docking the sulfonated mimetics onto the anionic-binding site I. Specifically, in this site, the sulphonyl-ethyl-sulfate group at one end of inhibitor **16** makes salt bridges and/or hydrogen bonds with Arg56. In contrast, the other end sulphonyl-ethyl-sulfate is in the near vicinity of Arg244. Other basic amino acids in the vicinity of the first sulphonyl-ethyl-sulfate group are Lys54, Lys61, and Lys68. One of the sulfonate groups attached to the naphthalene group makes salt bridges with Lys73 and Lys156, whereas the other sulfonate group on the naphthalene is in the near vicinity of Asn20.

Overall, the basic amino acid cluster of Lys54, Arg56, Lys61, Lys73, Arg244, and Arg303 residues may represent the putative binding site for the sulfonated heparin mimetics. This is to be experimentally determined by mutagenesis and X-ray crystallography studies. This site can be subsequently used to design more potent and selective inhibitors of human FXIIIa based on the structure of inhibitor **16**.

## Conclusion and Future Directions

In this study, we proposed that anion-binding site modulation of FXIIIa should be possible by targeting one or more of the anion-binding site(s) on the A subunit of FXIIIa. It was previously reported that heparin binds to FXIIIa with a *K*_D_ value of 2.4 μM,^36^ yet it does not inhibit its function. On the contrary, several sulfonated aromatic heparin mimetics inhibited FXIIIa with a range of IC_50_ values among which inhibitors **13** and **16** exhibit the most potent inhibition with IC_50_ values of 3.1 and 2.4 μM, respectively. Michaelis–Menten kinetics suggests that the inhibition by molecule **16** is unique since it only competes with the Gln-donor protein substrate i.e., dimethylcasein, but not with the Lys-donor small substrate i.e., dansylcadaverine (Figure 6 and Table 4), which further indicates that these molecules may bind to sites other than the active site. Given the nature of these molecules, a molecular modeling study was performed to elect a putative binding site; the resulting correlations between the docking scores and the biological parameters indicated that the sulfonated molecules preferentially bind to a cluster of Arg and Lys residues of anion-binding site I (Figure 9). Importantly, the inhibition by molecules **13** and **16** induced a concentration-dependent inhibition of FXIIIa–mediated fibrin polymerization, which indicates that they are physiologically relevant (Figure 7). Among the two molecules, inhibitor **16** exhibited superior functional selectivity against thrombin, factor Xa, factor XIa (Table 2), rTG-2 (Figure 5A), and papain. Not only that but inhibitor **16** demonstrated no toxicity (Figure 5B) and did not affect the human plasma clotting times (Table 3), at a comparable concentration to FXIIIa-IC_50_.

Interestingly, inhibitor **16** carries only four sulfonate groups, while other molecules carry more sulfonate groups and yet they demonstrated weaker potency of FXIIIa inhibition or even no inhibition at all. For example, UFH carries on average 75 negative charges and although it binds to FXIIIa, it does not inhibit its catalytic activity. The flavonoid trimers **1** and **2** possess 11 sulfonate groups; however, they are at least 15-fold less potent than inhibitor **16**. Likewise, all benzamide-based inhibitors **3**–**10** possess a greater number of sulfonate groups, yet they are at least 32-fold less potent than inhibitor **16** (Table 1). Importantly, the nonactive or less potent sulfonated molecules have a globular shape (inhibitors **9** and **10**) or a linear rigid shape (UFH and inhibitors **2**–**8**). Accordingly, linear flexible structures with fewer sulfonate groups such as inhibitors **11**–**19** demonstrated better FXIIIa inhibition potency. Indeed, the heightened flexibility exhibited by inhibitor **16** potentially enhances the likelihood of the molecule adopting optimal three-dimensional orientations of the sulfonate and aromatic moieties, which are more effectively recognized by the anion-binding site(s) of human FXIIIa. This underscores the notion that minor structural disparities exert a considerable influence on the potency of sulfonated heparin mimetic inhibitors targeting FXIIIa. Consequently, it suggests that future medicinal chemistry endeavors should prioritize optimizing the positioning of sulfonate groups to facilitate increased potency alongside enhanced selectivity. The inclusion of aromatic/hydrophobic features in an FXIIIa ligand is presumed to be crucial. Sulfonated heparin mimetics, characterized by a combination of hydrophilic/negatively charged and aromatic/hydrophobic traits, exhibit notable functional distinctions from their prototype molecule, heparin. These disparities seem to stem from the interaction of these molecules not only with basic residues like Lys and Arg but also with nonbasic residues. This interaction facilitates the formation of an alternative network of interconnected residues, leading to a conformational change that disrupts catalytic activity. Accordingly, inhibitor **16** will be considered in subsequent studies of the structure–activity relationship to optimize its potency and selectivity. Inhibitor **16** is also to be tested in human whole blood and other *in vivo* studies of venous thrombosis animal models to establish its *in vivo* anticoagulant activity.

Overall, the sulfonated aromatic heparin mimetics platform, particularly inhibitor **16**, presents a unique alternative approach to modulate FXIIIa.^58−61^ It appears to be very promising for providing molecules with potentially reduced bleeding risks. Furthermore, the use of specific molecules to perform molecular and cellular mechanistic studies may enhance our understanding of the role of FXIIIa in hemostasis. Finally, this work also suggests the possibility of using sulfonated aromatic heparin mimetics to modulate other transglutaminases.

## Materials and Methods

### Materials (Chemicals, Reagents, Enzymes, and Substrates)

Heparin mimetics **3**–**5** and **7**–**10** were synthesized, as reported earlier,^47,56,62−64^ whereas the rest of the mimetics were obtained from commercial vendors including Sigma Aldrich and Fisher Scientific. The sodium salt of mimetics **3**–**5** and **7**–**10** were obtained following modified protocols.^65^ For instance, the pure sodium salts were produced by SP Sephadex-Na cation exchange chromatography and then by Sephadex G10 size exclusion chromatography. Final products were obtained following 24 freeze-drying processes. The molecules were characterized by ^1^H NMR and ^13^C NMR, and the chemical shifts were similar to the reported values.^47,56,62−64^ The NMR spectrum was recorded in DMSO-*d*_6_ on a Bruker 600 MHz NMR spectrometer. The molecules’ purity was more than 95%. Representative NMR spectra for inhibitors **3**, **4**, **5**, and **10** are given in the Supporting Information.

Human plasma proteins such as FXIIIa, thrombin, factor Xa, and factor XIa were procured from Haematologic Technologies. Recombinant human TG-2 was sourced from Tocris Bioscience. Their activities were validated according to the supplier’s instructions. Chromogenic substrates for thrombin and factor Xa were acquired from Biomedica Diagnostics, while the factor XIa chromogenic substrate (S-2366) was obtained from Diapharma. Dithiothreitol, *N*,*N*-dimethylcasein, and dansylcadaverine were purchased from Sigma Aldrich. Stock solutions of thrombin and factor XIa were prepared in 50 mM Tris-HCl buffer, pH 7.4, containing 150 mM sodium chloride, 0.1% PEG8000, and 0.02% Tween80. The stock solution of factor Xa was prepared in 20 mM Tris-HCl buffer, pH 7.4, containing 100 mM sodium chloride, 0.1% PEG8000, 2.5 mM CaCl_2_, and 0.02% Tween80. The stock solutions of FXIIIa and rTG-2 were prepared in 50 mM Tris-HCl, 1 mM CaCl_2_, 100 mM sodium chloride, 0.02% Tween80, 0.1% PEG8000, and 2 mg/mL dimethylcasein. Papain and its chromogenic substrate (*N*-α-benzoyl-l-arginyl-4-nitroanilide) were obtained from Sigma Aldrich. Papain was prepared in 50 mM Tris-HCl buffer, pH 7.4, containing 100 mM NaCl, 100 mM DTT, 2.5 mM CaCl_2_, 0.1% PEG8000, and 0.02% Tween80. Human plasma for coagulation assays was obtained from George King Bio-Medica for the clotting assays. Thromboplastin-D (PT reagent), APTT reagent containing ellagic acid, and 0.025 M solution of CaCl_2_ were obtained from Thermo Fisher Scientific. Results were reproduced at least three times.

### Chemical Synthesis and Chemical Characterization

Anhydrous organic solvents and various reagents, including benzoyl chlorides, activated charcoal, and sulfonated aromatic amines, were procured from Fisher (Pittsburgh, PA) and utilized without further purification. Analytical thin-layer chromatography (TLC) was carried out employing UNIPLATETM silica gel GHLF 250 um precoated plates (ANALTECH, Newark, DE). Column chromatography was carried out utilizing silica gel (200–400 mesh, 60 Å) from Sigma Aldrich. All reactions were executed using oven-dried glassware. Flash chromatography was performed utilizing the Teledyne ISCO (Lincoln, NE) Combiflash RF system and disposable normal silica cartridges with particle sizes of 30–50 μ, mesh sizes of 230–400, and pore sizes of 60 Å. NMR data were reported in terms of chemical shift (ppm), signal multiplicity (s = singlet, d = doublet, t = triplet, q = quartet, dd = doublet of doublet, m = multiplet), coupling constants (Hz), and integration. Schematic presentation for the synthesis of heparin mimetics **3**–**5** and **7**–**10** is given in Scheme S1A–D.

**General procedure for amidation “method a”:** The sulfonated starting material was dissolved in water, and the pH was adjusted to 3–4. To this stirred solution, the benzoyl-chloride derivative dissolved in toluene was gradually added. The pH of the reaction mixture was controlled by periodically adding sodium carbonate solution. The reaction proceeded at room temperature overnight. Subsequently, the toluene layer was separated from the aqueous phase. The aqueous layer was then subjected to multiple washes with diethyl ether. Water was removed using a high vacuum pump. The crude product was purified through recrystallization from alcohol. **General procedure for aromatic nitro reduction “method b”:** In a stirring aqueous solution of the sulfonated-nitro derivative from the preceding step, Pd (10%) on charcoal (∼5% of the reactant’s weight) was introduced. The reaction mixture was subjected to hydrogenation at room temperature, and the reaction was allowed to proceed overnight. Subsequently, the catalyst was filtered off. The product was obtained by evaporating the water using a high vacuum pump. **General procedure for dimerization “method c”:** In a stirring aqueous solution of a sulfonated aromatic amine derivative, a solution of phosgene (15% in toluene) was gradually introduced at room temperature. The pH of the reaction mixture was kept at 3–4 by adding sodium carbonate solution. The aqueous phase was subsequently neutralized with sodium carbonate solution, and water was evaporated under vacuum. Excess salt was eliminated by stirring in alcohol (or by using a desalting column), as the product was insoluble in alcohol.

### Inhibition of Human FXIIIa in Bisubstrate, Fluorescence-Based, Transglutaminase Assay by Heparin Mimetics

To evaluate the effect of sulfonated molecules on human FXIIIa, a bisubstrate, fluorescence-based trans-glutamination assay was performed as we reported previously.^36,49^ Generally, 1 μL of the sulfonated molecule was diluted with 87 μL of pH 7.4 buffer (50 mM Tris-HCl, 1 mM CaCl_2_, 100 mM NaCl, and 2 mg/mL *N*,*N-*dimethylcasein) and 5 μL dithiothreitol (20 mM) at 37 °C followed by the addition of 2 μL of human FXIIIa (0.3 μM) and incubation for 10 min. The activity of FXIIIa was monitored following the addition of 5 μL of dansylcadaverine (2 mM) by measuring the initial rate of increase in fluorescence emission (λ_Ex._ = 360 nm and λ_Em._ = 550 nm). Relative residual FXIIIa activity at each concentration of the inhibitor was calculated from the ratio of FXIIIa activity in the presence and absence of the inhibitor. Logistic eq 1 was used to fit the concentration dependence of residual FXIIIa activity to obtain the potency (IC_50_) and efficacy (Δ*Y%*) of inhibition.
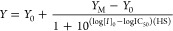
1

This equation defines *Y* as the ratio of residual FXIIIa activity with the inhibitor present compared to its absence. *Y*_M_ represents the maximum possible value of residual FXIIIa activity, while *Y*_O_ denotes the minimum possible value. IC_50_ signifies the concentration of the inhibitor causing 50% enzyme activity inhibition, and HS represents the Hill slope. Determination of *Y*_M_, *Y*_O_, IC_50_, and HS values is achieved through nonlinear curve fitting of the experimental data.

### Selectivity Studies Against Other Clotting Factors, Tissue Transglutaminase-2, and Papain

The inhibitory potential of various heparin mimetics against other clotting factors, including thrombin, factor Xa, and factor XIa, was also examined using established chromogenic substrate hydrolysis assays.^47,50^ For instance, in each well of a 96-well microplate containing either 85 or 185 μL of 20–50 mM Tris-HCl buffer (pH 7.4), supplemented with 100–150 mM NaCl, 0.1% PEG8000, and 0.02% Tween80 at either 25 or 37 °C, 5 μL of the inhibitor (or high purity water) and 5 μL of the enzyme were added. The final concentrations of the enzymes were 6 nM (thrombin), 1.09 nM (factor Xa), and 0.8 nM (factor XIa). After a 5 min incubation period, 5 μL of Spectrozyme TH (final concentration: 0.050 mM), Spectrozyme FXa (0.125 mM), or factor XIa substrate (0.35 mM) was swiftly added, and the residual enzyme activity was gauged from the initial rate of increase in absorbance at λ_405_ nm. The relative residual enzyme activity was calculated as a function of the inhibitor concentration. If 50% or more of the enzyme was inhibited, the results were then plotted using eq 1 to determine the corresponding IC_50_ values.

For tissue TG-2, a recombinant protein (rTG-2) was used in a bisubstrate, fluorescence-based trans-glutamination assay involving dansylcadaverine and *N*,*N*-dimethylcasein as substrates and under similar conditions used for human FXIIIa above. The activity of rTG-2 was monitored following the addition of 5 μL of dansylcadaverine (2 mM) by measuring the initial rate of increase in fluorescence emission (λ_Ex._ = 360 nm and λ_Em._ = 490 nm). Relative residual rTG-2 activity at each concentration of inhibitor **16** was calculated from the ratio of rTG-2 activity in the presence and absence of the inhibitor. Logistic eq 1 was used to fit the concentration dependence of residual rTG-2 activity to obtain the IC_50_ and Δ*Y*% of inhibition.

Direct inhibition of papain, a cysteine protease, by inhibitor **16** was also evaluated using a chromogenic substrate assay on a microplate reader as reported earlier.^36^ Briefly, to each well of a 96-well microplate containing 91 μL of 50 mM Tris-HCl buffer, pH 7.4, containing 100 mM NaCl, 2.5 mM CaCl_2_, 100 mM DTT, 0.1% PEG8000, and 0.02% Tween80 was added 5 μL of inhibitor **16** (0–20 mM) or vehicle, and 3 μL of papain (130 U/mL) at 37 °C. After 5 min incubation, 1 μL of *N*-α-benzoyl-l-arginine 4-nitroanilide hydrochloride, the chromogenic substrate (17.7 mM) was rapidly added, and the residual enzyme activity was measured from the initial rate of increase in absorbance at 405 nm. Relative residual enzyme activity as a function of the inhibitor concentration was measured and inhibition parameters were calculated using logistic eq 1.

### Effect of Heparin Mimetics on the Clotting of Normal Human Plasma

Clotting times, including activated partial thromboplastin time (APTT) and prothrombin time (PT), were assessed in normal human plasma utilizing the BBL Fibrosystem fibrometer from Becton–Dickinson, as detailed in previous publications.^51−54^ For the PT assay, thromboplastin-D was dissolved in 4 mL of highly purified water and then warmed to 37 °C. Subsequently, 10 μL of the inhibitor was added to 90 μL of normal human plasma and incubated for 30 s. Following this, 200 μL of prewarmed PT reagent was added, and the clotting time was recorded. In the APTT assay, 10 μL of the inhibitor was combined with 90 μL of normal human plasma and 100 μL of prewarmed APTT reagent (0.2% ellagic acid). After incubation for 4 min at 37 °C, clotting was initiated by adding 100 μL of prewarmed 0.025 M CaCl_2_, and the clotting time was documented. Multiple concentrations of each molecule were employed in both assays to establish a concentration vs effect curve. These data were then fitted to a quadratic trend line, enabling the determination of the concentration of the molecule required to double the plasma clotting time.

### The Cellular Toxicity of Inhibitors **13** and **16** (Effect on Cell Viability)

HEK-293 human embryonic kidney cells,^66^ CaCo-2 heterogeneous human epithelial colorectal adenocarcinoma cells,^67^ and MCF-7 human breast carcinoma cells^68^ were treated as reported previously.^69,70^ This procedure, conducted by the CMB core of XULA’s RCMI Program, employed a modified method utilizing Alamar Blue (resazurin) fluorescent dye. HEK-293, CaCo-2, and MCF-7 cells were cultured in 96-well plates under optimized conditions to achieve logarithmic growth. Throughout the experiment, the plates were maintained covered to ensure sterility. Following the addition of Alamar Blue dye to each well, the plates were incubated at 37 °C for 2 h. Subsequently, fluorescence readings (λ_Ex._ = 560 nm and λ_Em._ = 590 nm) were taken using the Synergy H1 multiplate reader (BioTek) to quantify cell numbers both in the absence (utilizing distilled water as a control) and the presence of inhibitors **13** and **16** (at a concentration of 10 μM; incubated for 3 days). The inclusion of a distilled water control served to validate the observed effects attributable to the inhibitors. Each measurement was conducted in quadruplicate to ascertain standard deviations.

### Effect of Heparin Mimetics on FXIIIa-Mediated Fibrin(ogen) Polymerization

The impact of inhibitors **13** and **16** on FXIIIa-mediated fibrin polymerization was further explored via gel electrophoresis, as previously described.^36,49^ A solution comprising 1.75 mg/mL fibrinogen and 0.9 μg/mL FXIIIa in 50 mM Tris-HCl buffer at pH 7.4 containing 10 mM CaCl_2_ was incubated with various concentrations of inhibitor **13** (5, 50, 500, 1000, 2000, and 3000 μM) and inhibitor **16** (5, 50, 500, 1000, 1500, and 2000 μM), and then subjected to clotting in the presence of human α-thrombin (1.25 μg/mL). The resulting clots were incubated for 24 h at room temperature before the addition of denaturing buffer containing 25 mM NaH_2_PO_4_, 1.9% (w/v) SDS, 5.7 M urea, and 1.9% (w/v) DTT, followed by overnight incubation at room temperature. Subsequently, the samples were boiled in a water bath for 10 min, followed by centrifugation at 12,000*g* at 20 °C for 3 min; the supernatants were then analyzed by SDS-PAGE on homogeneous 10% cross-linked gels and stained with Coomassie Brilliant Blue.

### Michaelis–Menten Kinetics of Dansylcadaverine and *N*,*N*-Dimethylcasein Conjugation Rate by Human FXIIIa

The initial rate of FXIIIa-mediated conjugation between dansylcadaverine and *N*,*N*-dimethylcasein was determined by monitoring the linear increase in fluorescence at λ_EM_ = 550 nm (λ_EX_ = 360 nm). This initial rate was assessed as a function of various concentrations of either (1) dansylcadaverine (ranging from 0 to 15 mM; resulting effective concentrations in the well were 0–750 μM) at a fixed saturating concentration of dimethylcasein (5 mg/mL), or (2) dimethylcasein (ranging from 0 to 15 mg/mL) at a fixed concentration of dansylcadaverine (5 mM; resulting effective concentration of 250 μM), both in the absence or presence of inhibitor **16** (at concentrations of 0, 0.5, 2.5, 15, and 25 μM). These assays were conducted in 50 mM Tris-HCl buffer at pH 8.0, containing 100 mM NaCl, 1 mM DTT, and 10 mM CaCl_2_ at 37 °C. The obtained data were fitted using the standard Michaelis–Menten equation (eq 2) to determine the values of *K*_M_ and *V*_MAX_.
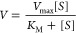
2

### Molecular Modeling Studies

The crystal structure of Factor XIII in complex with a peptide ligand bound to the active site (4KTY.pdb)^27^ was used. After removing all the crystal water molecules, protein structure preparation was carried out using the Molecular Operating Environment (MOE) 2020 software, and the assignment of the protonation state in the protein was set to pH 7 using Protonate 3D in MOE. All the molecules were energy minimized before being subjected to the docking studies. Three sites were considered for the docking experiment: the active site including the Cys314, the anion-binding site I (Lys54, Arg56, Lys61, Lys73, Arg244, and Arg303), and the anion-binding site II (Arg252, Lys257, Arg260, and Lys269). The protein–ligand docking calculations at each binding site were accomplished using the MOE software using the ASE scoring function. The ligand poses were ranked according to the docking scores.

## Supporting Information Available

The rationale for exploiting the sulfonated heparin mimetics to inhibit human FXIIIa (Figure S1); effect of increasing the concentration of inhibitor **16** on the kinetic parameters of dimethylcasein (Figure S2); effect of increasing the concentration of inhibitor **16** on the kinetic parameters of dansylcadaverine (Figure S3); schematic presentation for the synthesis of heparin mimetics **3**–**5** and **7**–**10** (Scheme S1); representative NMR data for molecules **3**, **4**, **5**, **10**, and **16** (PDF)
