# Supplementary material for: Discovery of Heparin Mimetic, Potent, and Selective Inhibitors of Human Clotting Factor XIIIa
Source: ACS Omega. 2024 Jul 3;9(28):31105–19. doi: 10.1021/acsomega.4c04518 (PMC11256326; doi:10.1021/acsomega.4c04518)
Supplement: Supplementary file 1 — ao4c04518_si_001.pdf [file ao4c04518_si_001.pdf]

Supplementary Information

Discovery of Heparin Mimetic, Potent, and Selective Inhibitors of Human Clotting Factor XIIIa

Kayla T. Vu,[1] Srabani Kar,[1] Navneet Goyal,[2] Madhusoodanan Mottamal,[2] Daniel K Afosah,[3] Rami A. Al-Horani*[1]

[1]*Division of Basic Pharmaceutical Sciences, College of Pharmacy, Xavier University of Louisiana, New Orleans LA 70125*

[2]*Department of Chemistry, Xavier University of Louisiana, New Orleans, LA 70125*

[3]*Department of Medicinal Chemistry, School of Pharmacy, Virginia Commonwealth University, Richmond, VA 23219*

* Address for correspondence: Dr. Rami A. Al-Horani, 1 Drexel Drive, College of Pharmacy, New Orleans, LA 70125-1089. Phone: (504) 520-7603, Fax: (504) 520-7954, Email: ralhoran@xula.edu

<u>Table of content</u>

Figure S1. The rationale for exploiting the sulfonated heparin mimetics to inhibit human FXIIIa.

Figure S2. Effect of increasing the concentration of inhibitor **16** on the kinetic parameters of dimethylcasein.

Figure S3. Effect of increasing the concentration of inhibitor **16** on the kinetic parameters of dansylcadaverine.

Scheme S1. A schematic presentation for the synthesis of heparin mimetics **3-5** and **7-10** is given in scheme S1 A-D.

NMR of Molecule 3

NMR of Molecule 4

NMR of Molecule 5

NMR of Molecule 10

Characterization of Inhibitor 16

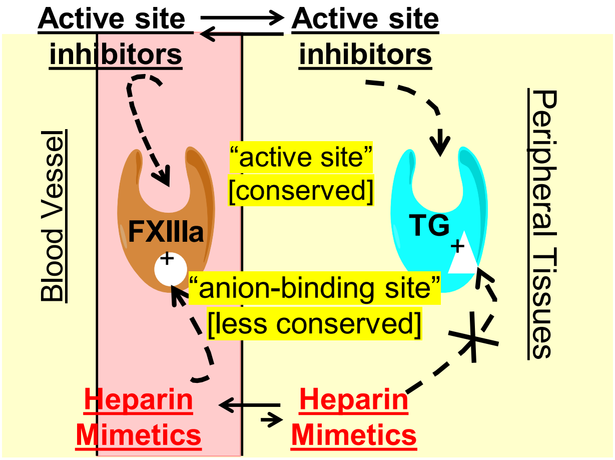

Figure S1. The rationale for exploiting the sulfonated heparin mimetics to inhibit human FXIIIa. These mimetics are proposed to demonstrate pharmacodynamic as well as pharmacokinetic selectivity which is very important for designing safer therapeutics. They are expected to target putative anionic-binding site(s) on FXIIIa which are believed to be less conserved than the active site in the transglutaminase family (FXIIIa vs other TGs), resulting in functional selectivity. Given their anionic characteristics, the sulfonated heparin mimetics are more likely to be confined to the vascular system inhibiting FXIIIa and not distributing to peripheral tissues or the central nervous system where are TGs, resulting in distribution selectivity.

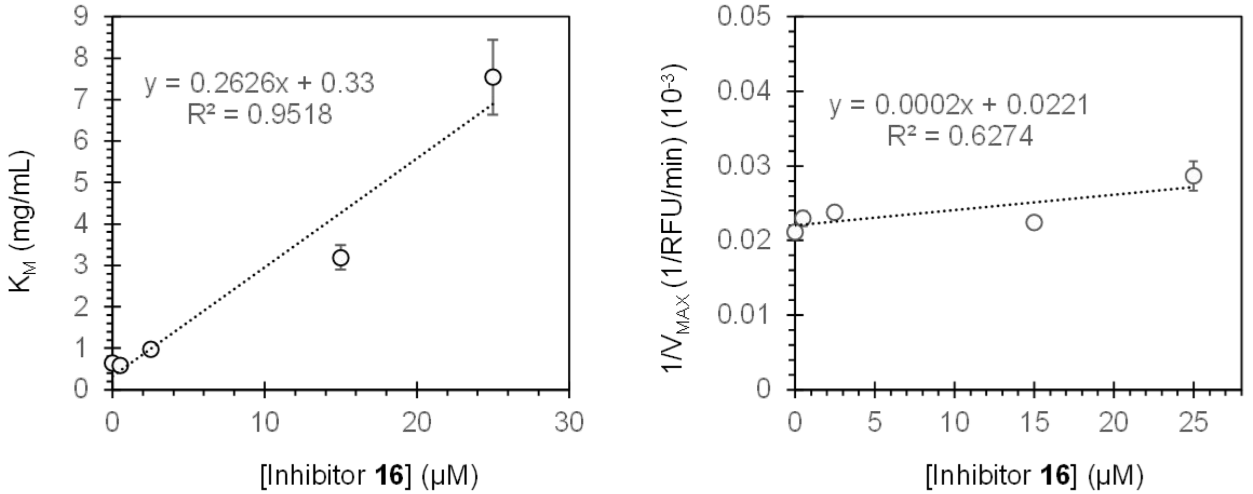

Figure S2. Effect of increasing the concentration of inhibitor **16** on the kinetic parameters of dimethylcasein.

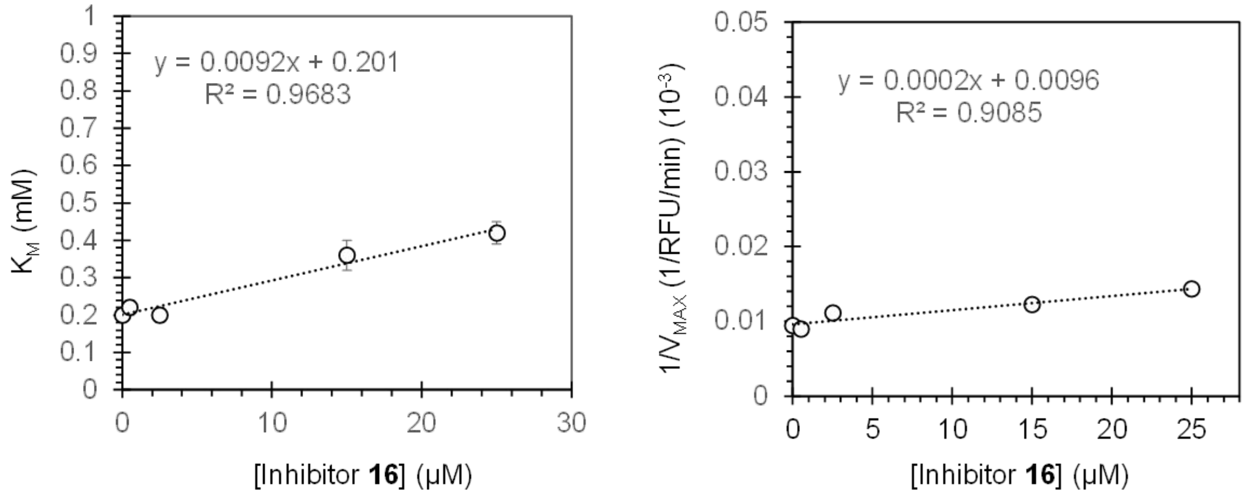

Figure S3. Effect of increasing the concentration of inhibitor **16** on the kinetic parameters of dansylcadaverine.

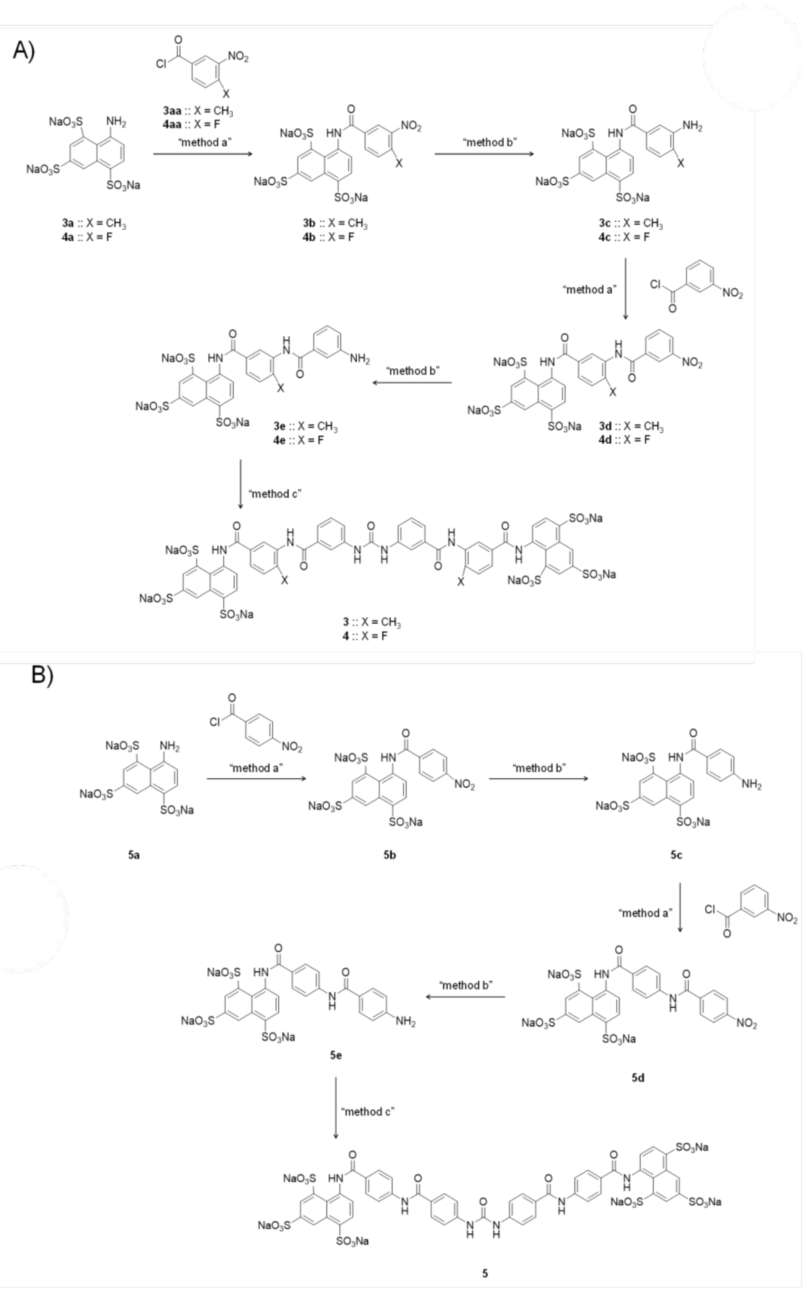

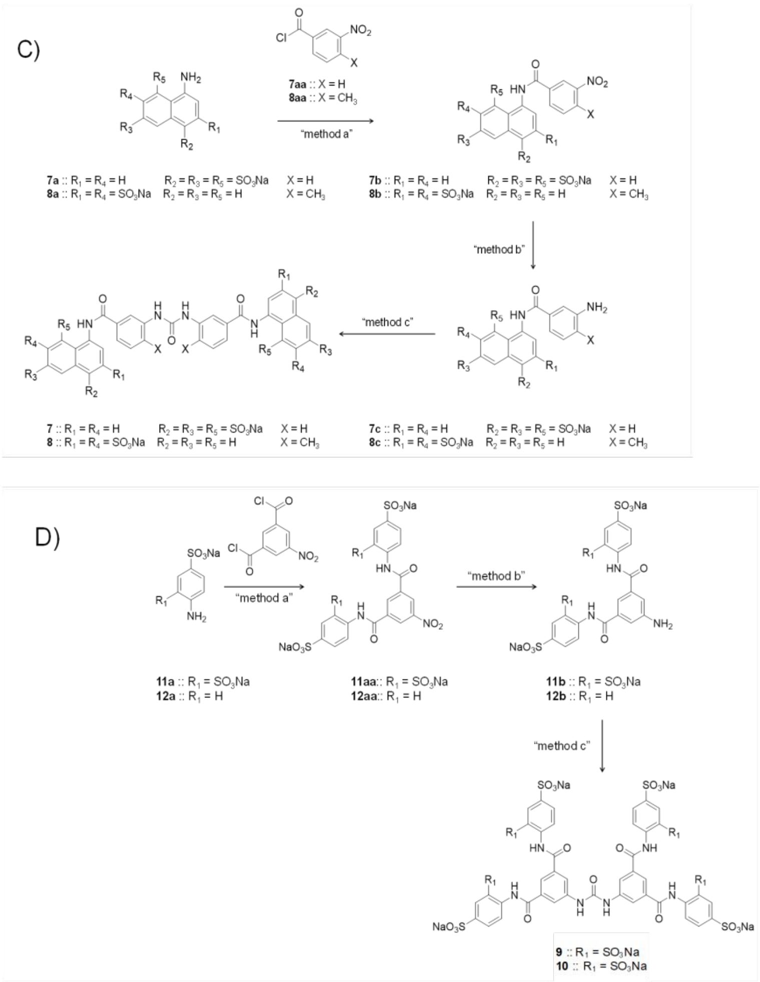

Scheme S1. A) Synthesis of molecules **3** and **4**. B) Synthesis of molecule **5**. C) Synthesis of molecules **7** and **8**. D) Synthesis of molecules **9** and **10**. "Method a": H_2O, pH 4, RT, overnight (benzyl chloride derivative is in toluene), yield 40-65%; "Method b": 10% Pd/activated charcoal, H_2O, RT, overnight, yield ~90%; "Method c": $COCl_2$ solution (15 wt. %) in toluene, H_2O, pH 3.5, RT, overnight, yield 30-50%.

NMR of Molecule 3

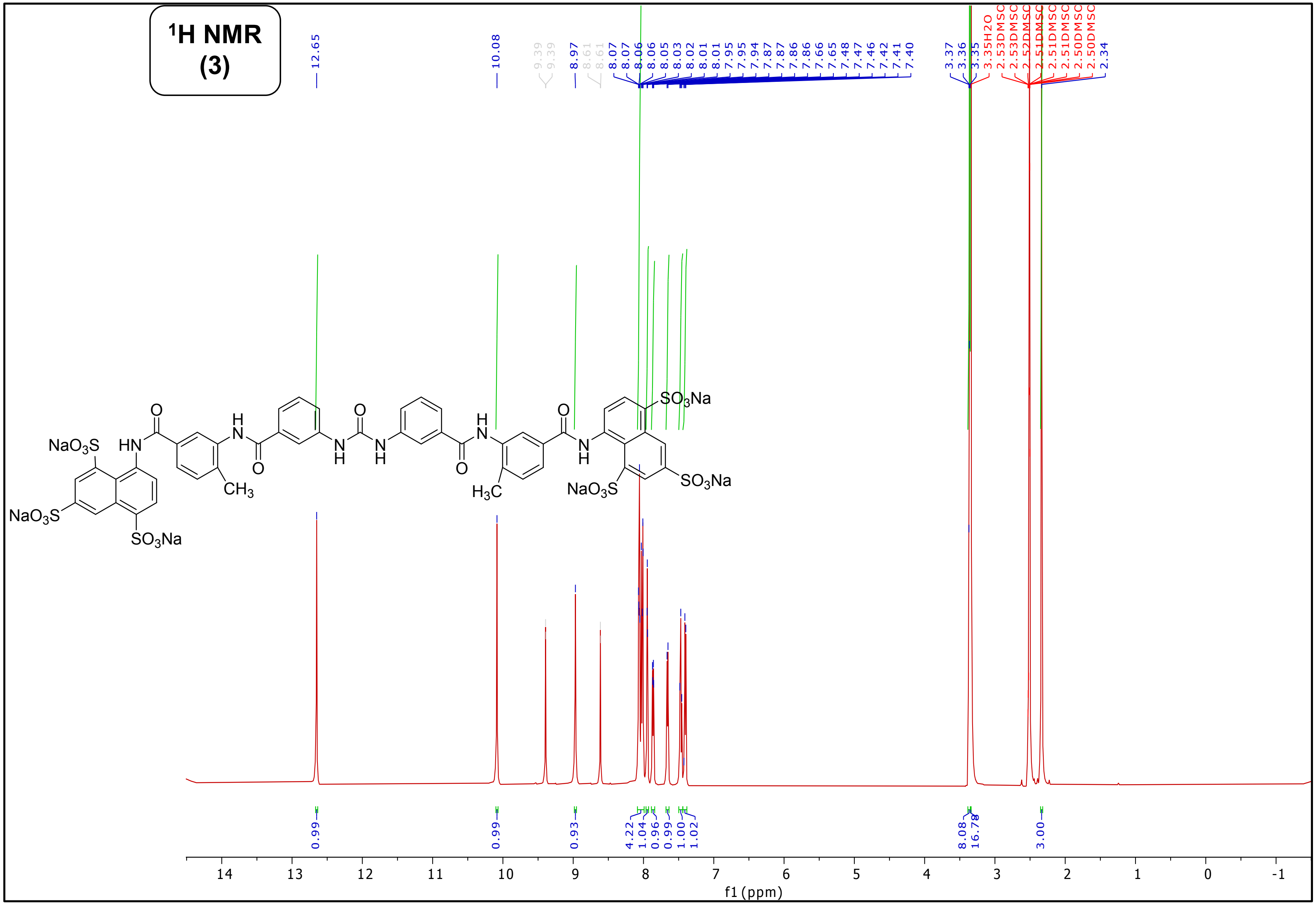

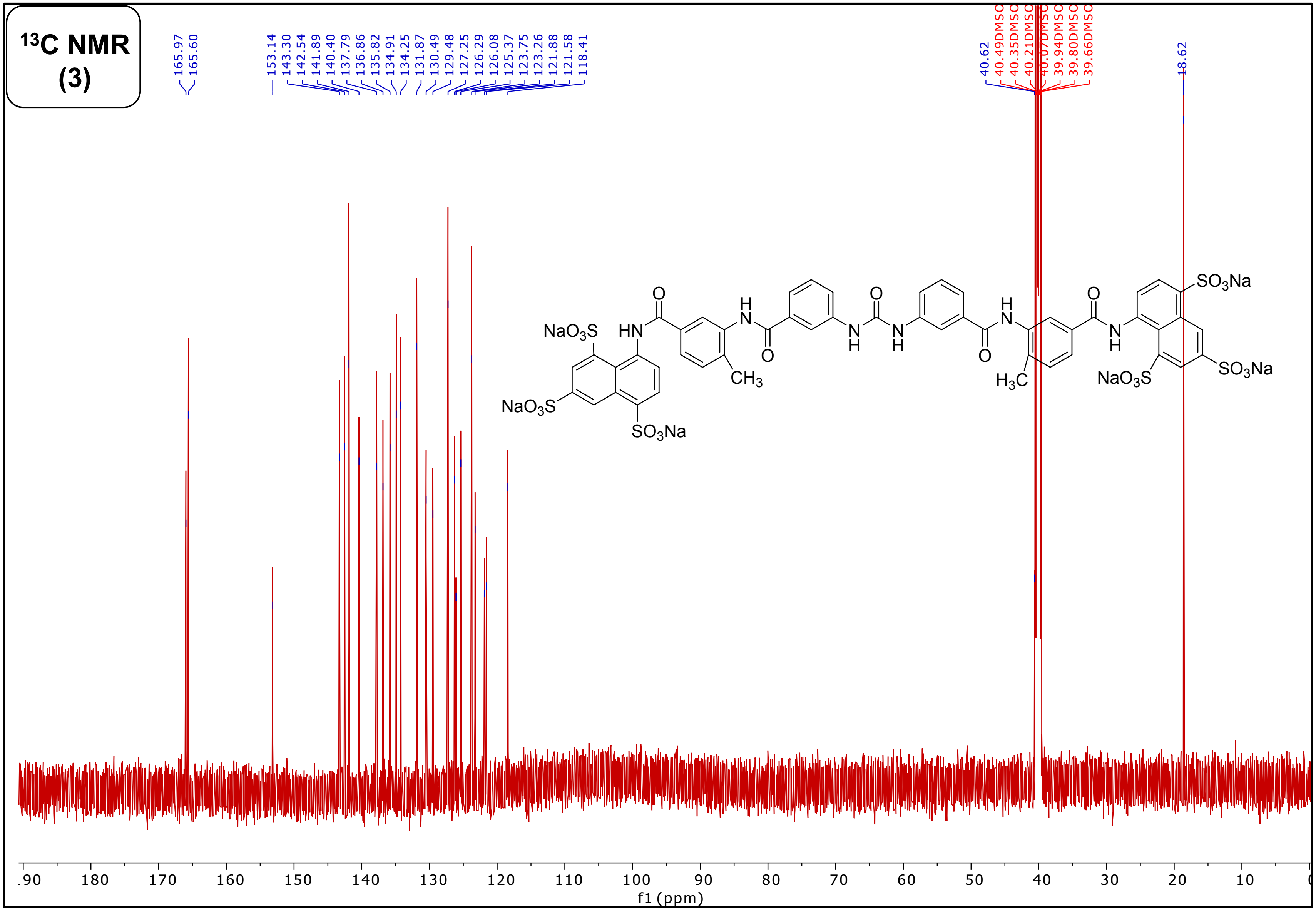

13C NMR
(3)
165.97
165.60
153.14
143.30
142.54
141.89
140.40
137.79
136.86
135.82
134.91
134.25
131.87
130.49
129.48
127.25
126.29
126.08
125.37
123.75
123.26
121.88
121.58
118.41
40.62
40.49DMSO
40.35DMSO
40.21DMSO
40.07DMSO
39.94DMSO
39.80DMSO
39.66DMSO
18.62
f1 (ppm)

NMR of Molecule 4

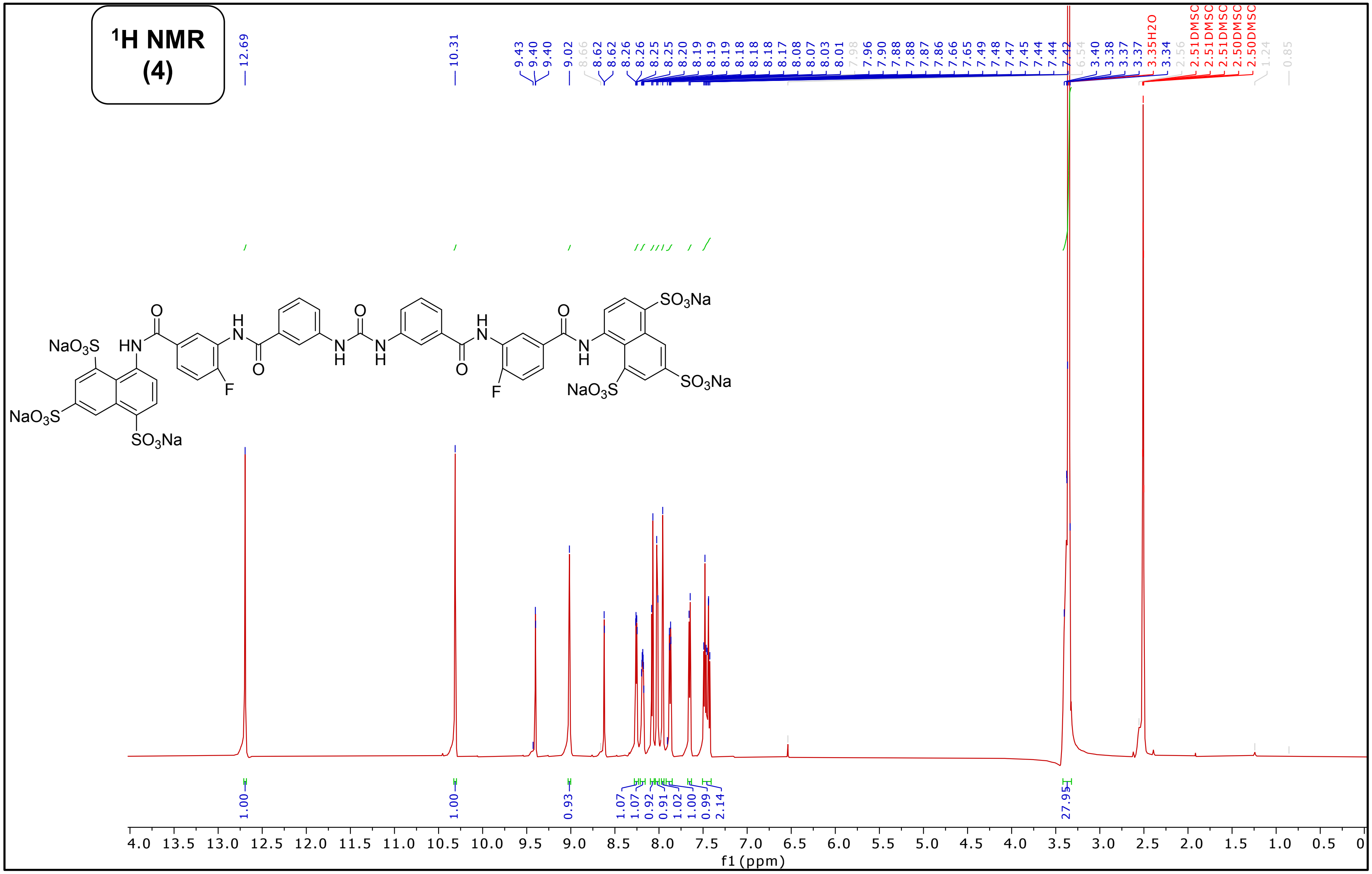

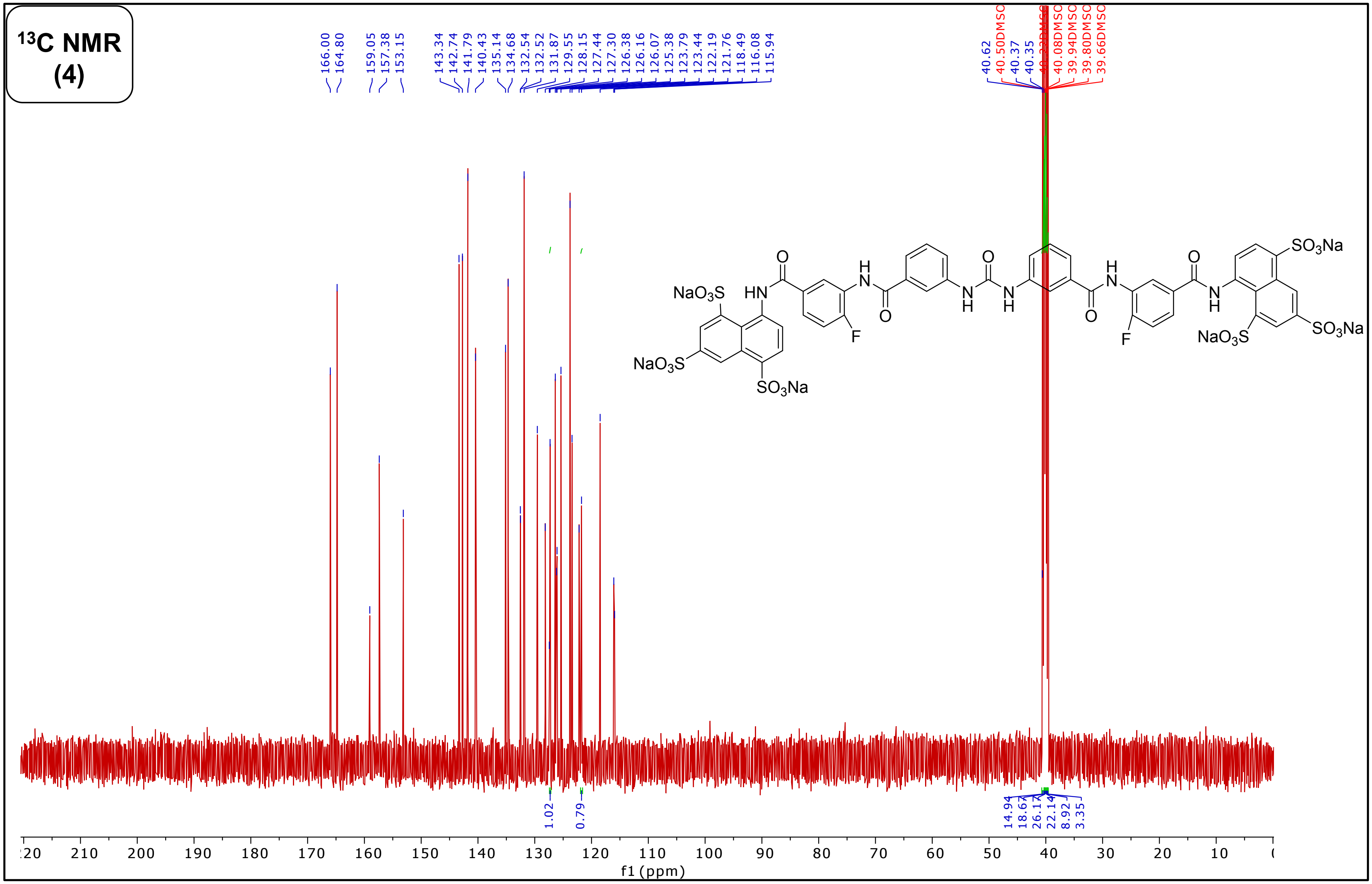

NMR of Molecule 5

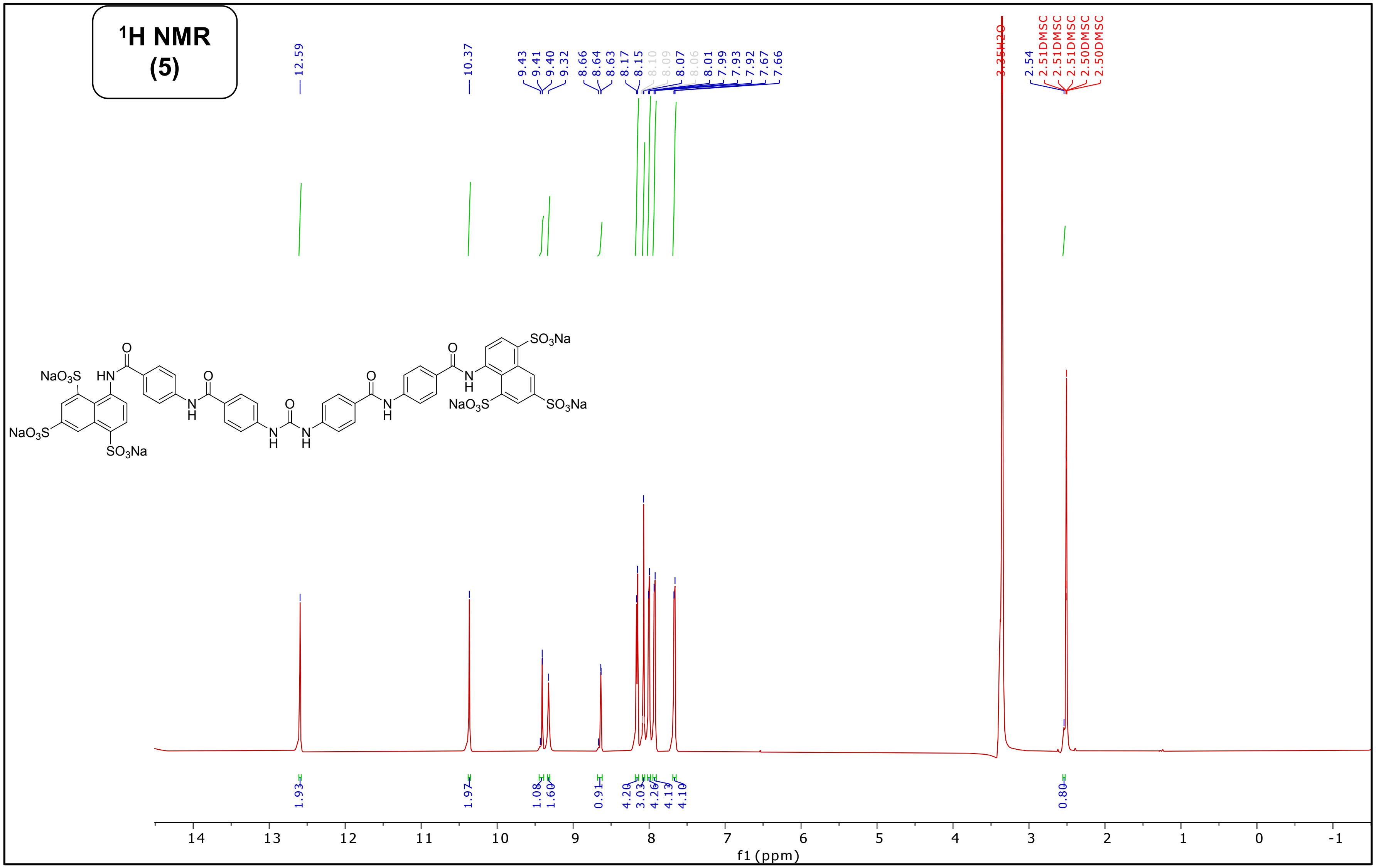

¹H NMR
(5)
12.59
10.37
9.43
9.41
9.40
9.32
8.66
8.64
8.63
8.17
8.15
8.10
8.09
8.07
8.06
8.01
7.99
7.93
7.92
7.67
7.66
3.35H2O
2.54
2.51DMSO
2.51DMSO
2.51DMSO
2.50DMSO
2.50DMSO
1.93
1.97
1.08
1.60
0.91
4.20
3.03
4.26
4.13
4.10
0.80
14
13
12
11
10
9
8
7
6
5
4
3
2
1
0
-1
f1 (ppm)
NaO3S
HN
O
N
H
O
N
H
O
N
H
O
N
H
O
N
H
SO3Na
NaO3S
SO3Na
NaO3S
SO3Na

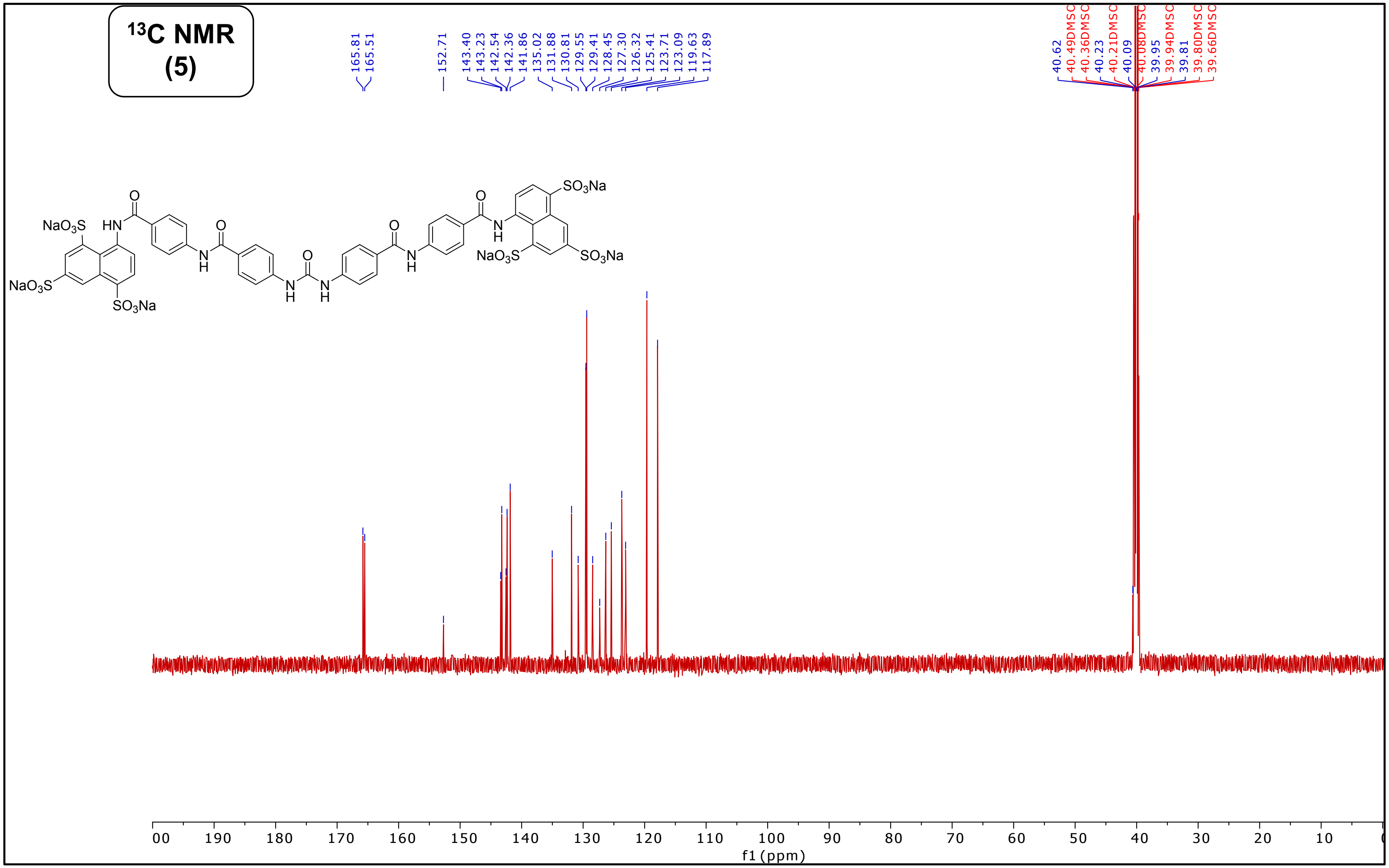

NMR of Molecule 10

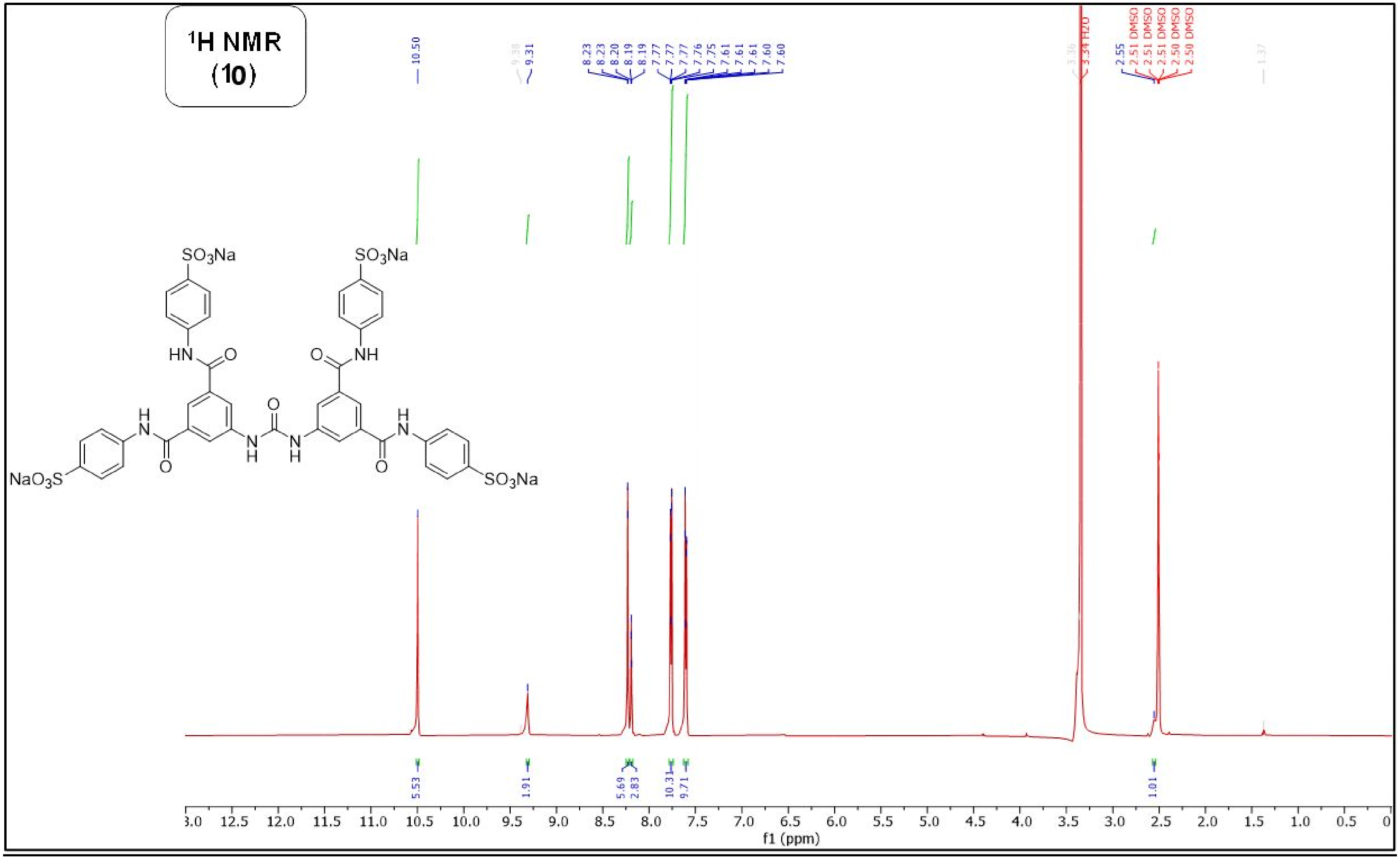

1H NMR
(10)
SO3Na
HN
O
NH
NaO3S
f1 (ppm)

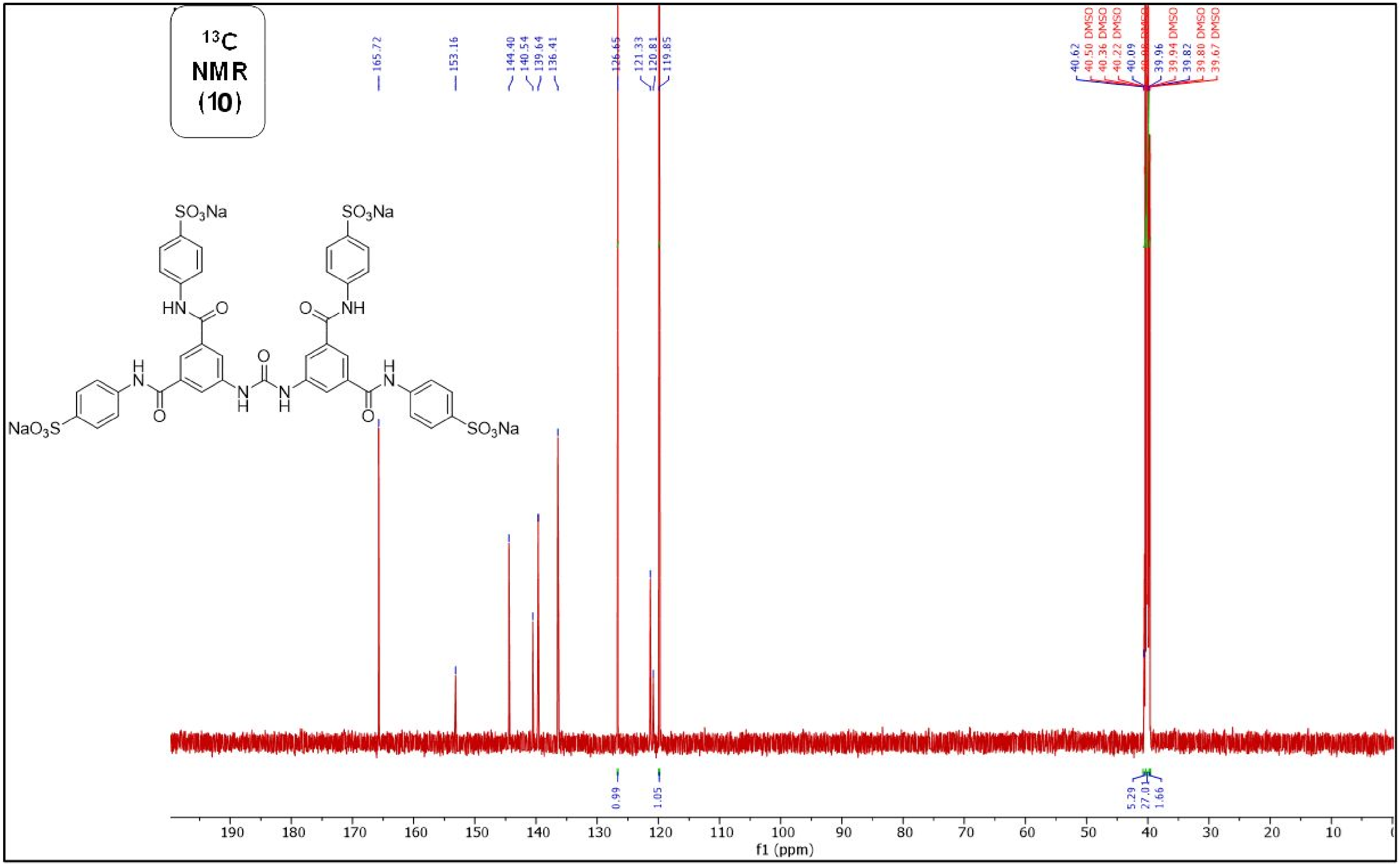

13C NMR (10)
165.72
153.16
144.40
140.54
139.64
136.41
126.65
121.33
120.81
119.85
40.62
40.50 DMSO
40.36 DMSO
40.22 DMSO
40.09
39.96
39.94 DMSO
39.82
39.80 DMSO
39.67 DMSO
SO3Na
HN
NH
O
N
H
NaO3S
0.99
1.05
5.29
27.01
1.66
190
180
170
160
150
140
130
120
110
100
90
80
70
60
50
40
30
20
10
f1 (ppm)

Characterization of Inhibitor 16

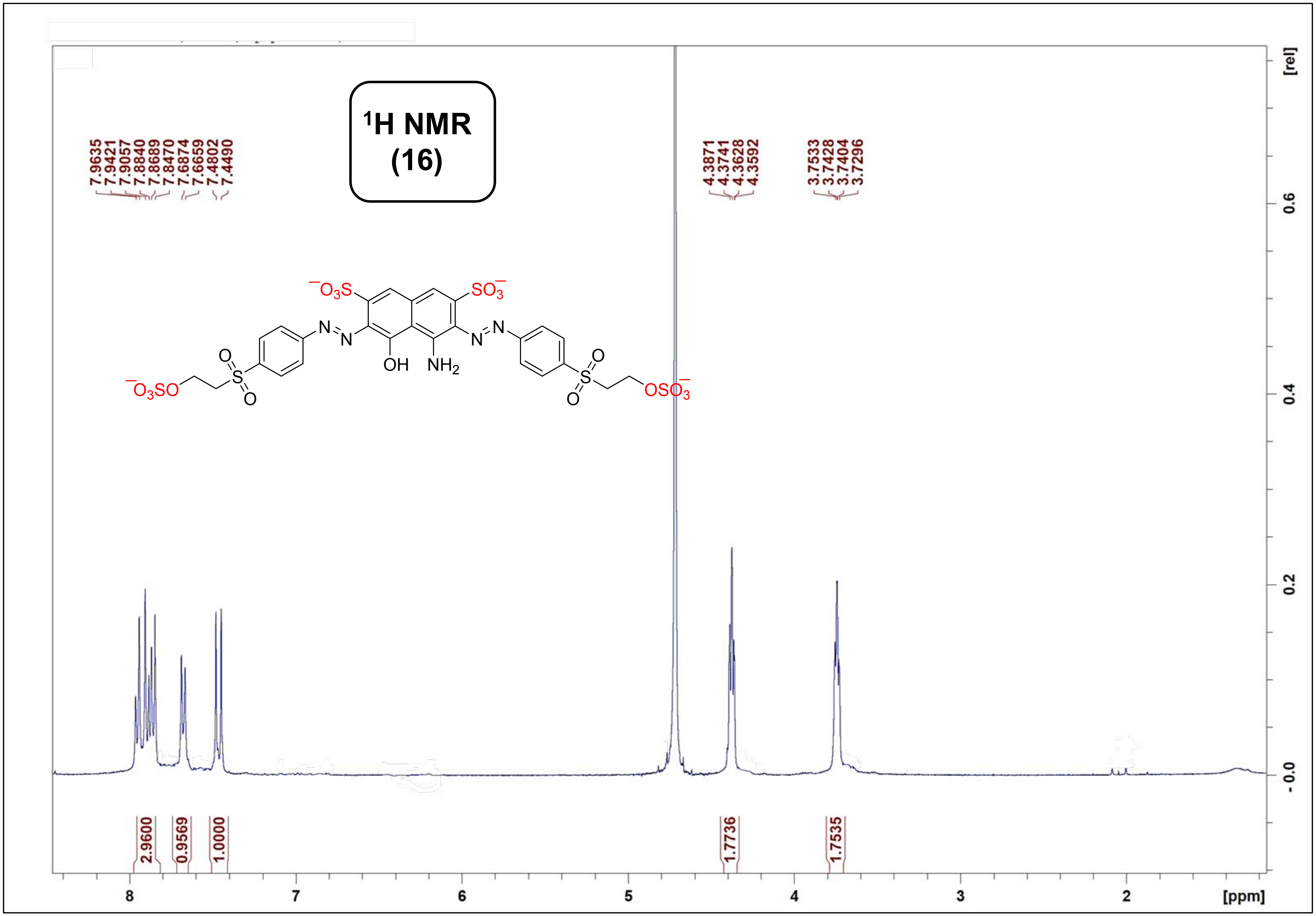

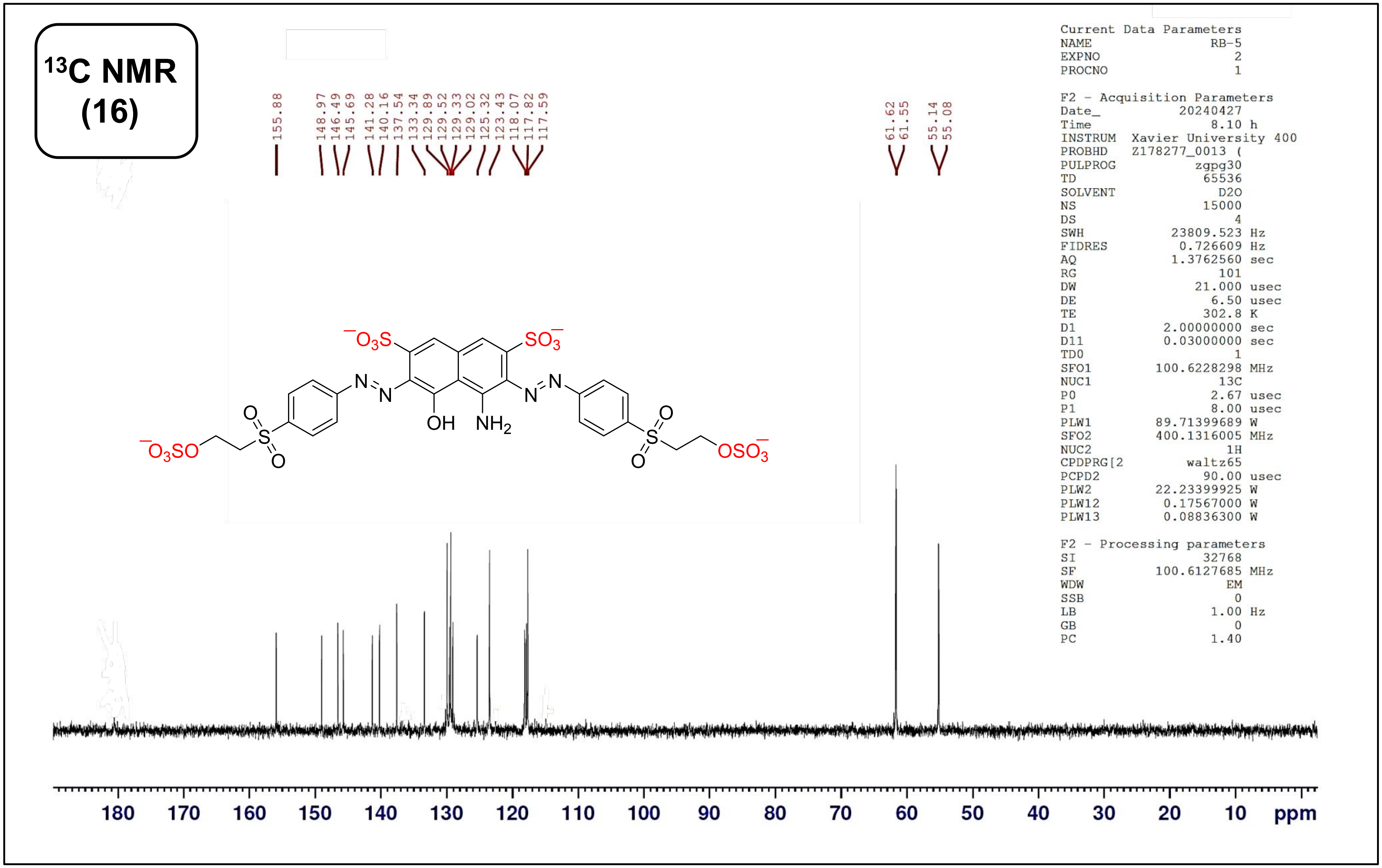
^{13}C NMR
(16)
155.88
148.97
146.49
145.69
141.28
140.16
137.54
133.34
129.89
129.52
129.33
129.02
125.32
123.43
118.07
117.82
117.59
61.62
61.55
55.14
55.08
Current Data Parameters
NAME RB-5
EXPNO 2
PROCNO 1
F2 - Acquisition Parameters
Date_ 20240427
Time 8.10 h
INSTRUM Xavier University 400
PROBHD Z178277_0013 (
PULPROG zgpg30
TD 65536
SOLVENT D2O
NS 15000
DS 4
SWH 23809.523 Hz
FIDRES 0.726609 Hz
AQ 1.3762560 sec
RG 101
DW 21.000 usec
DE 6.50 usec
TE 302.8 K
D1 2.00000000 sec
D11 0.03000000 sec
TD0 1
SFO1 100.6228298 MHz
NUC1 13C
P0 2.67 usec
P1 8.00 usec
PLW1 89.71399689 W
SFO2 400.1316005 MHz
NUC2 1H
CPDPRG[2 waltz65
PCPD2 90.00 usec
PLW2 22.23399925 W
PLW12 0.17567000 W
PLW13 0.08836300 W
F2 - Processing parameters
SI 32768
SF 100.6127685 MHz
WDW EM
SSB 0
LB 1.00 Hz
GB 0
PC 1.40
180 170 160 150 140 130 120 110 100 90 80 70 60 50 40 30 20 10 ppm

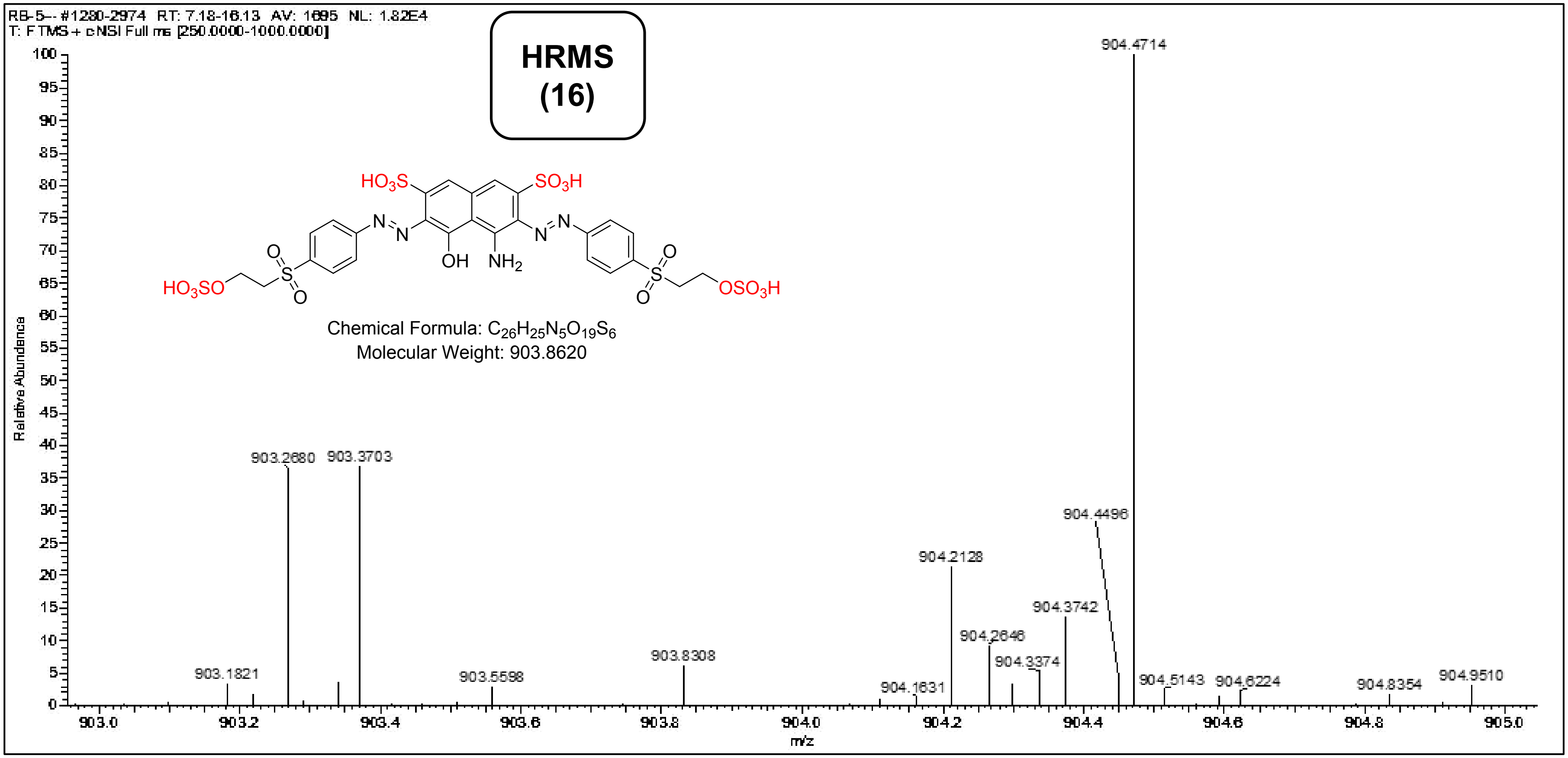
RB-5-- #1280-2974 RT: 7.18-16.13 AV: 1695 NL: 1.82E4
T: FTMS + c NSI Full ms [250.0000-1000.0000]
HRMS
(16)
HO3S
SO3H
OH NH2
HO3SO
OSO3H
Chemical Formula: C26H25N5O19S6
Molecular Weight: 903.8620
Relative Abundance
m/z
904.4714
903.2680
903.3703
904.4496
904.2128
904.3742
904.2646
904.3374
903.8308
903.1821
903.5598
904.1631
904.5143
904.6224
904.8354
904.9510
